# Non-closed acoustic cloaking devices enabled by sequential-step linear coordinate transformations

**DOI:** 10.1038/s41598-021-81331-3

**Published:** 2021-01-19

**Authors:** Zahra Basiri, Mohammad Hosein Fakheri, Ali Abdolali, Chen Shen

**Affiliations:** 1 Applied Electromagnetic Laboratory, School of Electrical Engineering, Iran University of Science and Technology, 1684613114 Tehran, Iran; 2 Department of Mechanical Engineering, Rowan University, Glassboro, NJ 08028 USA; 3 Department of Electrical and Computer Engineering, Duke University, Durham, NC 27708 USA

**Keywords:** Acoustics, Metamaterials, Transformation optics

## Abstract

Hitherto acoustic cloaking devices, which conceal objects externally, depend on objects' characteristics. Despite previous works, we design cloaking devices placed adjacent to an arbitrary object and make it invisible without the need to make it enclosed. Applying sequential linear coordinate transformations leads to a non-closed acoustic cloak with homogeneous materials, creating an open invisible region. Firstly, we propose to design a non-closed carpet cloak to conceal objects on a reflecting plane. Numerical simulations verify the cloaking effect, which is completely independent of the geometry and material properties of the hidden object. Moreover, we extend this idea to achieve a directional acoustic cloak with homogeneous materials that can render arbitrary objects in free space invisible to incident radiation. To demonstrate the feasibility of the realization, a non-resonant meta-atom is utilized which dramatically facilitated the physical realization of our design. Due to the simple acoustic constitutive parameters of the presented structures, this work paves the way toward realization of non-closed acoustic devices, which could find applications in airborne sound manipulation and underwater demands.

## Introduction

Invisibility cloak is one of the most attractive research topics due to its exotic properties in deflecting the waves around objects. A powerful approach to achieve invisibility devices is the coordinate transformation method firstly utilized by Pendry et al. for the design of ideal electromagnetic (EM) cloak^1^. Not long after, Cummer and Schurig presented a two-dimensional (2D) acoustic cloak by illustrating the analogy between 2D time harmonic Maxwell equations and acoustic wave equations^2^. Later, a similar coordinate transformation method was further extended to design of 3D acoustic cloaks by Chen and Chan^3^. The same relations through the acoustic scattering theory were also derived in^4^ by Cummer et al. Due to the potential applications of cloaking in a variety of both acoustics and electromagnetics scenarios, numerous schemes have been devoted to theoretical development and fabrication of cloaking devices^5–10^. The major difficulty in ideal cloak implementation is the requirement of extreme material properties, which is resulted from the mapping of a point or line to the cloaked region in the cylindrical or spherical cloak, respectively. To remove this obstacle, the concept of carpet cloak was proposed^11^. Carpet cloak or ground plane cloak is a device that restores the signature of the target as if the incident wave reflects from a mirror plane. The carpet cloak is designed by employing a coordinate transformation from a flat sheet to the cloaked region that creates a bump with the mirror plane, resolving the need to extreme materials’ parameters. Later, inspired by the carpet cloak concept, unidirectional free space cloak was proposed to represent the cloaking effect for a specified direction of propagation^12^. The strategy to unidirectional cloak design is based on the property of the mirror plane to be invisible when probed by a plane wave propagating parallel to it.

The first proposal of carpet cloak and unidirectional cloak was presented through quasi-conformal mapping^11,12^ with the remarkable advantage of minimizing the anisotropy of obtained materials^12–15^. However, the inhomogeneous structure of quasi-conformal cloaks leads to a difficult fabrication process and neglecting the weak anisotropy causes a lateral shift in the reflected wave^16^. Another disadvantage is the size of this type of cloaks, which is bulky compared to that of the target. To overcome these challenges, linear transformation based carpet cloak^17–20^ and unidirectional cloak^21–23^ were proposed. Cloaks based on linear coordinate transformation have homogeneous constitutive parameters with finite anisotropy, which obviates the need for space dependent materials. So far, in addition to huge amount of theoretical investigations to advance of cloaks^24–28^, several studies experimentally demonstrated acoustic carpet cloaks via homogeneous fluid-like materials. For example, in airborne acoustics, perforated plastic plates have been applied for 2D^29^ and 3D^30^ ground plane cloaks and for underwater acoustics, steal strips^31^ and brass plates^32^ have been proposed. In addition, acoustic unidirectional cloaks were implemented via composites of metals and porous materials for a multi-layered host medium^33^ and meta-fluid structured by slab-shape units for air host^34^.

Although considerable progress has been made in invisibility cloaks, all conventional cloaking devices are “interior” cloaks and thus prevent the target from interacting with outer world, which is a restriction for the use of these applicable devices. In order to obviate this drawback, Lai et al.^35^ presented "external" cloaking and illusion devices for the EM framework. Because of relevant acoustic demands, not long after the first proposal, the idea was extended to acoustics by Zhu et al.^36^. The key enabling feature of designing such external or “non-closed” invisibility devices is the complementary media concept, which is designed to conceal a predefined object^37–40^. However, complementary media based cloaks depend on the shape and material properties of the hidden object^41,42^. Therefore, any changes in description of object or any movements disturb the cloaking effect. Consequently, the dependence of the cloaking device on target and inevitable inhomogeneity of obtained materials are the remaining challenges that make the implementation of conventional external cloaks practically nonrealistic.

Differ from conventional design, in this paper; we design non-closed acoustic cloaks (NCACs), in which their materials are feasibly independent of the target geometry and its constitutive materials. The proposed technique is based on applying sequential linear transformations to create a non-closed invisible region positioned on a reflecting plane. Therefore, any standing or moving object in the hidden region will be acoustically invisible without being blinded. Due to the nature of linear coordinate transformation, the obtained materials of NCAC are homogeneous which results in easy to fabricate non-closed carpet cloaks. Moreover, the idea of non-closed devices is further extended to design a free space unidirectional acoustic cloak that can conceal any arbitrary objects. The full wave numerical simulations using COMSOL finite element solver verify the expected behavior of NCACs. Finally, to give a more realistic point of view, the required constitutive materials are realized with the aid of non-resonant acoustic meta-atoms.

## Design and theory

### Non-closed carpet cloak

**Figure 1:**
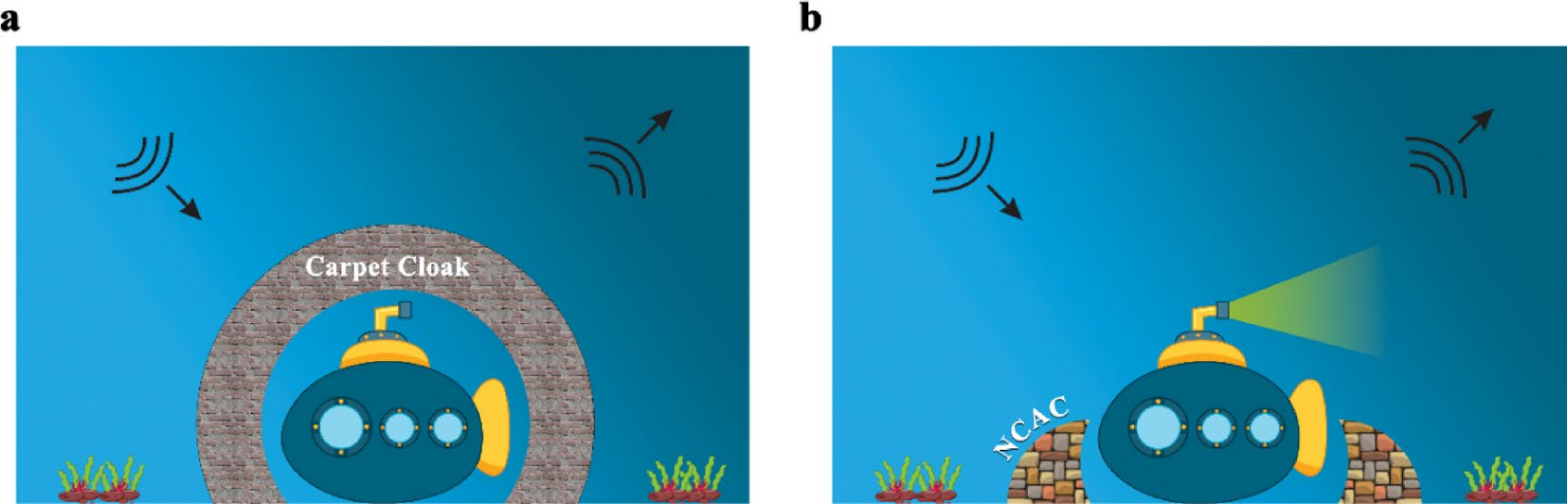
A schematic of (**a**) conventional carpet cloak and (**b**) NCAC for arbitrary object. The desired non-closed carpet cloak has an acoustically equal signature with its corresponding conventional one. So, the incident wave is reflected from the non-closed device as a reflecting plane, without any perturbation.

To start with a conception of idea, it is well understood that the conventional carpet cloak^18^ has a hidden region underneath a sound hard boundary bump where any arbitrary object located becomes invisible. The main purpose is to design an acoustic cloak, which, in addition to attempting to avoid enclosing objects, its constitutive parameters being independent of the material, geometry and position of objects. Figure 1 illustrates the equivalence between the behavior of the desired non-closed device and its corresponding conventional one, where the reflected wave of the whole NCAC and neighbor target is not disturbed.

**Figure 2:**
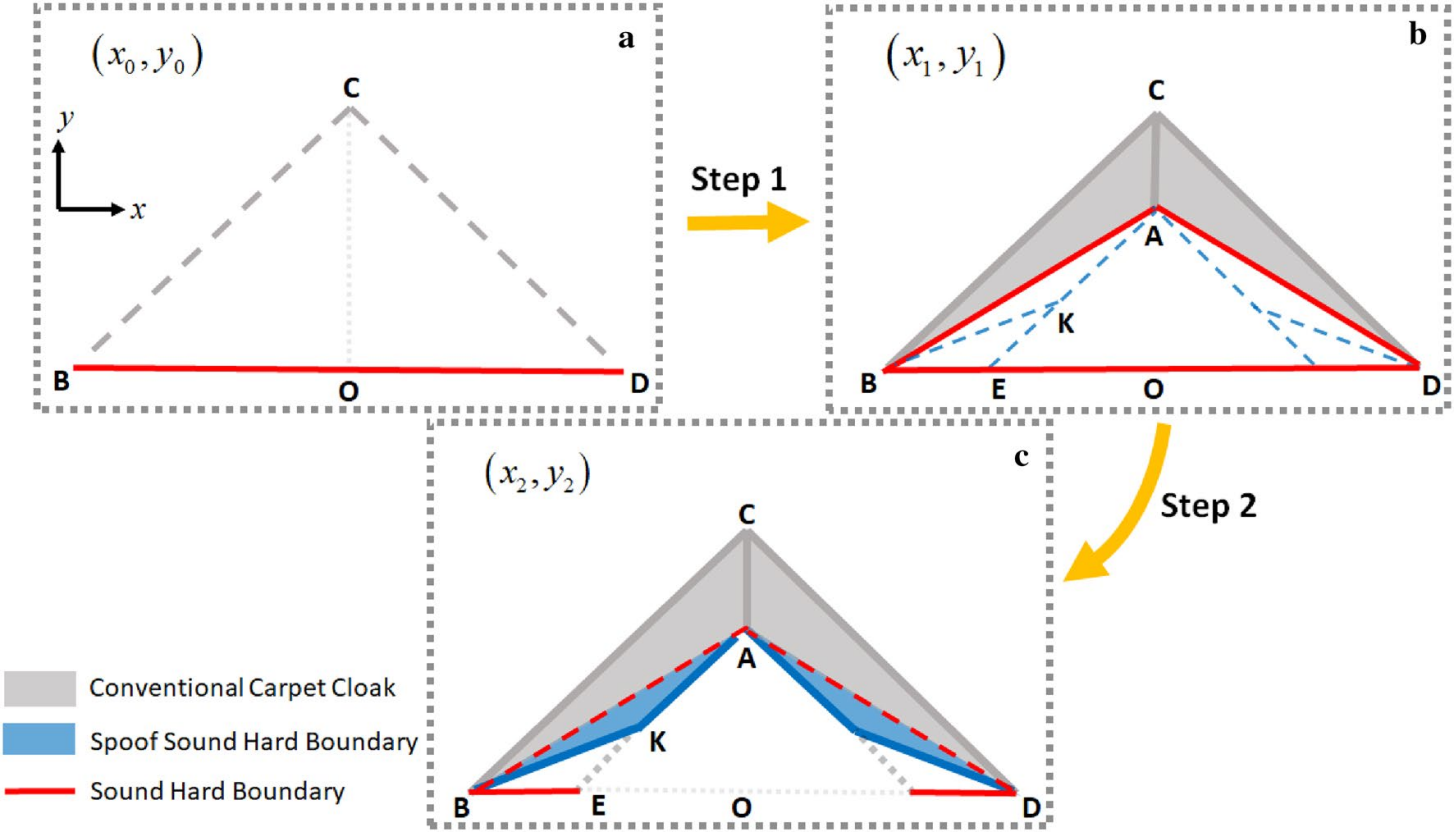
Schematic diagram of first and second steps to NCAC design. (**a**) The reference space. (**b**) Conventional carpet cloak's scheme. (**c**) The spoof sound hard boundary folds the ∆KBE region in (x_1_, y_1_) space to ∆KBE in (x_2_,y_2_) space and makes an illusionary sound hard boundary condition on $\overline{{{\text{AB}}}}$ boundary that is shown by red dashed lines.

At the first step, the conventional carpet cloak^18^ will be proposed. Without loss of generality, the problem is discussed in 2D framework while it could be extended to 3D case. As demonstrated in Fig. 2a,b, the triangle $\Delta BCD$ in reference space $\left( {x_{0} ,y_{0} } \right)$ is mapped to the quadrilateral $BCDA$ in real space $\left( {x_{1} ,y_{1} } \right)$. Therefore, any object located beneath the $BAD$ sound hard boundary (SHB) bump becomes invisible. At the second step, the existing SHB in the carpet cloak is physically eliminated by utilizing a linear folded transformation. The resulted folded medium is a type of complementary media^35^ and is actually an SHB mimicking structure, without any imposed sound hard boundary condition. Considering the geometrical symmetry of the scheme, the problem is discussed only in the left half space of the reference and real spaces and a same method is applied to the right side. Analytically, a linear coordinate transformation that maps $\Delta KBE$ region in the reference space $\left( {x_{1} ,y_{1} } \right)$ to $\Delta KBA$ region in the real space $\left( {x_{2} ,y_{2} } \right)$ folds the ground plane boundary $\overline{BE}$ to $\overline{AB}$ and makes an effective SHB on $\overline{AB}$, as shown in Fig. 2c. Hence, the folded medium creates an illusionary SHB bump and regenerates the cloaking effect of the conventional carpet cloak without making any disturbance in the scattered wave. From now on, for brevity, we call the domain mimicking the sound hard boundary condition as “spoof sound hard boundary” (SSHB) and the carpet cloak without physical SHB bump as the carpet cloak with SSHBs.

**Figure 3:**
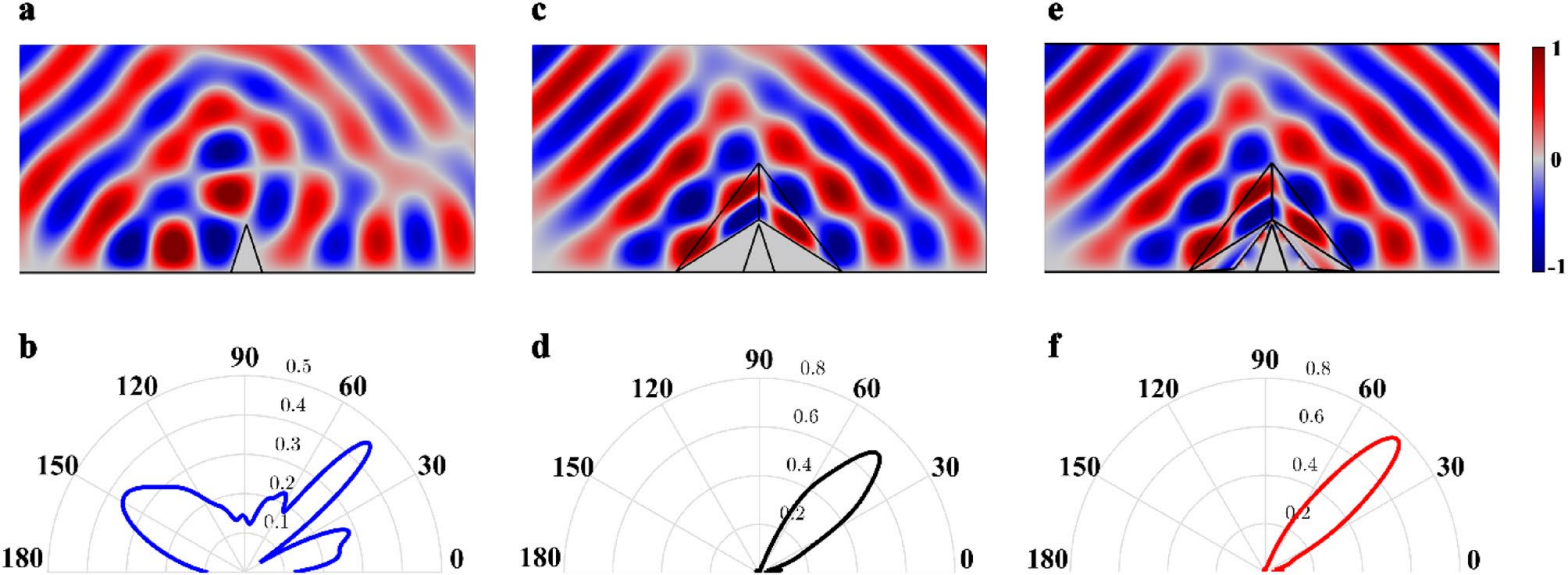
Comparison between the field distribution of target, conventional carpet cloak and the carpet cloak with SSHBs. (**a**,**b**) The near field distribution and far field scattering pattern of the object with sound hard boundaries. (**c**,**d**) The near field distribution and far field scattering pattern of conventional carpet cloak. (**e**,**f**) The near field distribution and far field scattering pattern of the carpet cloak with SSHBs. It can be seen that the results in (**c**,**d**) and (**e**,**f**) are closely similar to each other.

In order to verify the effect of carpet cloak with SSHBs, numerical simulations are carried out using the COMSOL Multiphysics finite element solver. All simulations are performed by adopting the acoustic pressure field in the frequency of 3.34 kHz, and the host medium is chosen as air with $\rho_{0} = 1\,\,kg/m^{3}$ and $c_{0} = 343\,\,m/s$ values for mass density and speed of sound, respectively. It is worth to mention that, due to the frequency independence of transformation acoustics method^1–3^, our design strategy is valid for any desired operating frequency and the chosen frequency, is completely arbitrary. By taking the left side of problem's geometry as the reference, at the first and second steps of the design, the acoustic constitutive materials for conventional cloaks and the SSHB region are $$\overline{\overline{\rho }}_{cloak} = \rho_{0} \left[ {\begin{array}{*{20}c} {1.2871} & { - 1.2146} \\ { - 1.2146} & {1.9231} \\ \end{array} } \right],\kappa_{cloak} = {1}{\text{.9231}}\kappa_{0}$$ and $$\overline{\overline{\rho }}_{SSHB} = \rho_{0} \left[ {\begin{array}{*{20}c} { - 16.0124} & {13.0663} \\ {13.0663} & { - 10.7247} \\ \end{array} } \right],\kappa_{SSHB} = { - 0}{\text{.0714}}\kappa_{0}$$, where $\rho_{0}$ and $\kappa_{0}$ densities and bulk modulus of the background medium, respectively. The geometric parameters and derivation of the acoustic properties are detailed in the next section. The performance of the carpet cloak with SSHBs comparing with the conventional carpet cloak is demonstrated in Fig. 3a–f. The near-field and far-field distribution of pressure fields of an object located on the reflecting plane is shown in Fig. 3a,b, respectively. It is obvious that the presence of object disturbs the reflecting wave. In Fig. 3e,f, the carpet cloak with SSHBs is used to make the object invisible, whose scattering is closely similar to that of the conventional carpet cloak depicted in Fig. 3c,d. All the aforementioned simulations verify the idea of the illusionary SHB bump and prove the validity of the carpet cloak with SSHBs.

**Figure 4:**
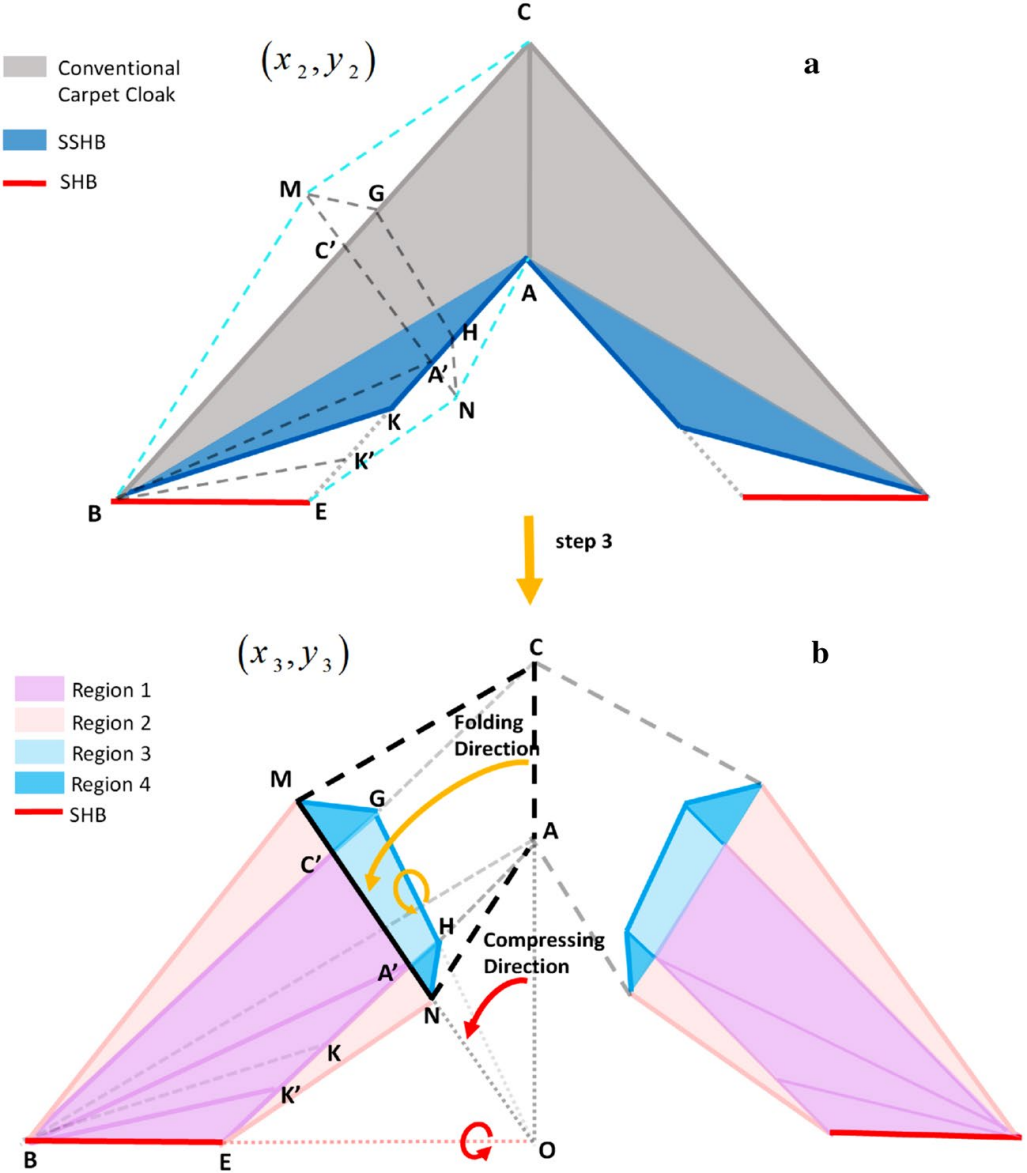
Schematic diagram of the proposed object independent non-closed carpet cloak. (**a**) Carpet cloak with SSHBs. In order to make a window on this structure, the trapezoidal region *AEBC *and its surrounding fluid (dashed light blue lines) in the reference space (x_2_,y_2_) are going to be compressed to smaller domains. (**b**) Non-closed carpet cloak. The trapezoidal domain *AEBC*, is compressed to the smaller purple trapezoid denoted by region 1 in real space (x_3_,y_3_) and the surrounding host medium, is also compressed to the melon domains named region 2. Blue domains show the complementary materials. The black dashed line boundaries represent the original boundaries before compressing. The incident wave that meeting the dashed lines, is routed to the solid lines by complementary domains denoted by regions 3 and 4. Significant result of this transformation is that the signature of the created window on conventional carpet cloak is canceled. Therefore, the whole structure is matched to host fluid and acts like an illusionary single reflecting plane.

Finally, we can open a window in the carpet cloak with SSHBs to make its hidden region non-closed. Subsequently, at the third step, the carpet cloak with SSHBs and its surrounding fluid in the reference space $\left( {x_{2} ,y_{2} } \right)$ (Fig. 4a), are compressed to smaller domains in the real space $\left( {x_{3} ,y_{3} } \right)$, as shown in Fig. 4b. For detail, a linear transformation that maps the $\Delta OCB$ region to the $\Delta OC^{\prime}B$, as illustrated by red arrows in Fig. 4b, compresses the carpet cloak with SSHBs to non-closed regions depicted by region 1. Similarly, the $\Delta BMC$ and $\Delta ENA$ regions of the surrounding host fluid are also compressed to $\Delta BMC^{\prime}$ and $\Delta ENA^{\prime}$ domains denoted by region 2 in order to satisfy the matching condition^35,43^. Thereupon, the compressing transformations map $\overline{NA}$, $\overline{AC}$ and $\overline{CM}$ boundaries in the reference space $\left( {x_{2} ,y_{2} } \right)$ to $\overline{{NA^{\prime}}}$, $\overline{{A^{\prime}C^{\prime}}}$ and $\overline{{C^{\prime}M}}$ boundaries in the real space $\left( {x_{3} ,y_{3} } \right)$.

In the coordinate transformation frame, by mapping the carpet cloak with SSHBs to the compressed regions, the path of the wave and outer boundaries of the structure in the reference space follow the compressing transformation in the real space and are mapped to transformed lines in compressed domains. In order to restore the path of wave, other folded regions are also utilized. The added domains, which are denoted by regions 3 and 4 shown in Fig. 4b, are a type of complementary media^35^ that convey the route of wave in the real space to the compressed space by employing a linear transformation. The linear coordinate mapping folds the black dashed lines representing the reference space to black solid lines in the real space. The significant result of this mapping is the invariance of waves’ path from the reference space to the real space (Fig. 4b). Another remarkable implication of complementary regions is mapping of each outer boundary to itself, which results in satisfaction of the matching condition^35,43^. Finally, the incident acoustic fields are conveyed to the compressed domains and track the compression direction, bypass the cloaked region and are scattered as that in the reference space.

To mathematically restate the complementary regions, firstly, the complementary medium $GHA^{\prime}C^{\prime}$ denoted by region 3 in Fig. 4b is obtained by mapping $\Delta OGC$ in the reference space to $\Delta OGC^{\prime}$ in the real space. In fact, region 3 folds the $\overline{AC}$ boundary in the reference space to $\overline{A^{\prime}C^{\prime}}$ in the real space and $\overline{GH}$ boundary to itself, as illustrated by yellow arrows in Fig. 4b. Similarly, the complementary media of the compressed surrounding host fluid, denoted by region 4 in Fig. 4b, are achieved by mapping $\Delta GMC$ and $\Delta HNA$ in the reference space, respectively to $\Delta GMC^{\prime}$ and $\Delta HNA^{\prime}$ in the real space. The obtained material of $\Delta GMC^{\prime}$ region folds $\overline{MC}$ to $\overline{MC^{\prime}}$ and $\overline{MG}$ to itself. In the same manner, the material of $\Delta HNA^{\prime}$ region folds $\overline{NA}$ to $\overline{{NA^{\prime}}}$ and $\overline{HN}$ to itself. In summary, the employed linear coordinate transformations to achieve complementary materials drawn by regions 3 and 4 are chosen in a way that $\overline{MC}$, $\overline{NA}$ and $\overline{AC}$ dashed line boundaries in the reference space, respectively fold to $\overline{MC^{\prime}}$, $\overline{NA^{\prime}}$ and $\overline{A^{\prime}C^{\prime}}$ solid line boundaries in the real space and all outer boundaries of the real space, i.e. $\overline{MG}$, $\overline{GH}$ and $\overline{HN}$ are mapped to themselves (Fig. 4b). As a result, the compressed structure in the presence of complementary materials is matched to the host medium. Therefore, the compounded compressed regions and complementary materials have an identical scattering effect with the conventional carpet cloak in the reference space or equally, with a reflecting plane.

By applying the compression and complementing transformations (the steps illustrated in Fig. 4), the necessitating homogeneous constitutive parameters are obtained for each region, which are given in Table 1. More detailed explanations and derivation of data presented in Table 1, have been proposed in Supplementary Material 1.

**Table 1:** The constitutive materials of the NCAC's regions.

Region 1	$$\overline{\overline{\rho }}_{{BA^{\prime}C^{\prime}}} = \rho_{0} \left[ {\begin{array}{*{20}c} {0.8703} & { - 0.8978} \\ { - 0.8978} & {2.0752} \\ \end{array} } \right],\kappa_{{BA^{\prime}C^{\prime}}} = 2.8441\kappa_{0}$$
	$$\overline{\overline{\rho }}_{{BK^{\prime}E}} = \rho_{0} \left[ {\begin{array}{*{20}c} {{0}{\text{.6762}}} & {{0}{\text{.2461}}} \\ {{0}{\text{.2461}}} & {{1}{\text{.5685}}} \\ \end{array} } \right],\kappa_{{BK^{\prime}E}} = {1}{\text{.4789}}\kappa_{0}$$
	$$\overline{\overline{\rho }}_{{BA^{\prime}K^{\prime}}} = \rho_{0} \left[ {\begin{array}{*{20}c} { - 10.8272} & {9.1255} \\ {9.1255} & { - 7.7837} \\ \end{array} } \right],\kappa_{{BA^{\prime}K^{\prime}}} = - 0.1056\kappa_{0}$$
Region 2	$$\overline{\overline{\rho }}_{BMC^{\prime}} = \rho_{0} \left[ {\begin{array}{*{20}c} {3.6440} & { - 0.0305} \\ { - 0.0305} & {0.2747} \\ \end{array} } \right],\kappa_{{BMC^{\prime}}} = {1}{\text{.4789}}\kappa_{0}$$
	$$\overline{\overline{\rho }}_{{ENA^{\prime}}} = \rho_{0} \left[ {\begin{array}{*{20}c} {0.4298} & { - 0.2741} \\ { - 0.2741} & {2.5015} \\ \end{array} } \right],\kappa_{{ENA^{\prime}}} = {1}{\text{.4789}}\kappa_{0}$$
Region 3	$$\overline{\overline{\rho }}_{{GHC^{\prime}A^{\prime}}} = \rho_{0} \left[ {\begin{array}{*{20}c} { - 5.3226} & {0.7788} \\ {0.7788} & { - 0.3018} \\ \end{array} } \right],\kappa_{{GHC^{\prime}A^{\prime}}} = - {1}{\text{.3897}}\kappa_{0}$$
Region 4	$$\overline{\overline{\rho }}_{{MC^{\prime}G}} = \rho_{0} \left[ {\begin{array}{*{20}c} { - 1.2121} & { - 0.3662} \\ { - 0.3662} & { - 0.9356} \\ \end{array} } \right],\kappa_{{MC^{\prime}G}} = - {1}{\text{.3897}}\kappa_{0}$$
	$$\overline{\overline{\rho }}_{{NA^{\prime}H}} = \rho_{0} \left[ {\begin{array}{*{20}c} { - 7.6589} & {1.8469} \\ {1.8469} & { - 0.5759} \\ \end{array} } \right],\kappa_{{NA^{\prime}H}} = - {1}{\text{.3897}}\kappa_{0}$$

**Figure 5:**
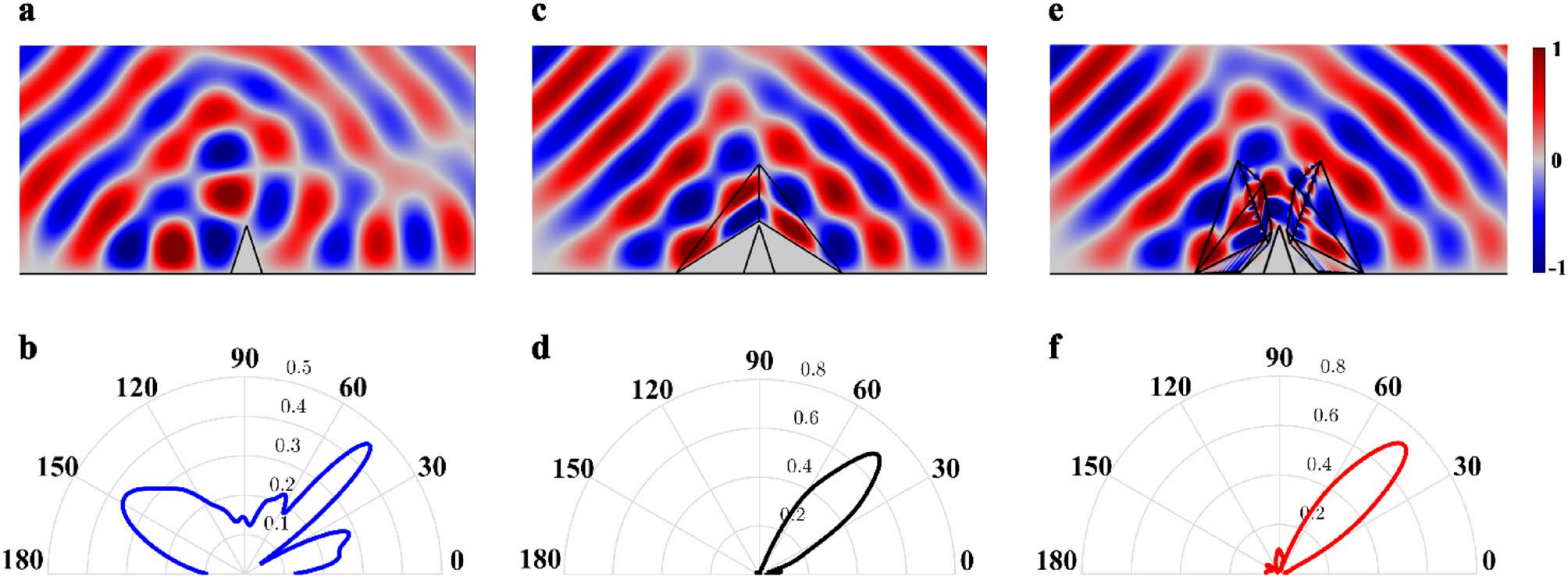
Simulation results of non-closed carpet cloak. (**a**,**b**) Near field distributions and far field scattering pattern of the target with sound hard boundaries that is located on the reflecting plane. The scattered field of reflecting plane is disturbed in the presence of the target. (**c**,**d**) Near field distributions and far field scattering pattern of the conventional carpet cloak. (**e**,**f**) Near field distributions and far field scattering pattern of the target near the non-closed carpet cloak. The non-closed device restored the scattering pattern of the object as well as the reflecting plane. It is also similar to field distribution of the conventional structure, which illustrates the validity of the fenestrated carpet cloak.

Recently, the acoustic metamaterial technology has been the subject of numerous researches^44–48^. These studies show promising results for realization of anisotropic media which are presented in Table 1. The detailed information of geometry and constructive materials is also supported in the next section. In order to provide more intuitive perception of the designed NCAC, numerical simulations are performed. In Fig. 5, the numerical simulation results are demonstrated to compare the scattering pattern of the object with and without the non-closed carpet cloak. Figure 5a,b respectively display the near-field distribution and far-field scattering pattern of an object with SHB boundaries under the excitation of a Gaussian beam propagating at the angle of $\pi /4$ which disturbs the reflected wave. As demonstrated in Fig. 5e,f, the presence of the designed NCAC adjacent to the object restores the pressure field distribution and far-field pattern the same as the conventional carpet cloak (shown in Fig. 5c,d). It is obvious that due to the presence of SSHB, the incident fields do not penetrate the hidden region. Because of this feature, the target shape and its constitutive materials do not affect the cloaking behavior of the non-closed structure. Therefore, the significant effect of the device is to hide an arbitrary object without making it blinded. The simulation results decisively confirm the identical behavior of the designed non-closed carpet cloak with the original carpet cloak. Using homogeneous materials is another supreme benefit that facilitates realization of non-closed invisibilities. It is worth mentioning that there is a tradeoff between the extent of the created window on the cloaking device and difficulty of the realization process. If the cloaking shell is compressed to a highly smaller region, it looks more fascinating and is really toward fictions; however, complementary materials take higher values of negative constitutive parameters and implementation of materials will become harder.

### Theories and coordinate transformations

In this section, we present mathematical derivation of the proposed sequential-step coordinate transformations to design of non-closed carpet cloak that is schematically illustrated in Fig. 4. For convenience, the left side of the structure is considered and all relations can be extended to the right side. The corresponding transformation equation for the first designing step, which represents the mapping of a reflecting plane in the reference space $\left( {x_{0} ,y_{0} } \right)$ to a SHB bump in the real space denoted by $\left( {x_{1} ,y_{1} } \right)$ (as depicted in Fig. 2), can be expressed as:

1 $$ \Delta BOC \to \Delta BAC:\begin{array}{*{20}c} {} & \begin{gathered} x_{1} = x_{0} \hfill \\ y_{1} = \frac{1}{{m_{AB} }}x_{0} + \left( {1 - \frac{{Y_{A} }}{{Y_{C} }}} \right)y_{0} + Y_{A} \hfill \\ \end{gathered} \\ \end{array} $$

wherein $Y_{A}$ and $Y_{C}$ are the ordinates of $A$ and $C$ points in Fig. 2 and $m_{AB}$ is the inverted slope of $\overline{AB}$ or ${{\left( {X_{A} - X_{B} } \right)} \mathord{\left/ {\vphantom {{\left( {X_{A} - X_{B} } \right)} {\left( {Y_{A} - Y_{B} } \right)}}} \right. \kern-\nulldelimiterspace} {\left( {Y_{A} - Y_{B} } \right)}}$. Generally, each point is determined as $\left( {X_{i} ,Y_{i} } \right)$ where the subscript of i denotes the points' name. Moreover, the parameter “m” denotes the inverted slope of the line labeled with its index. Due to the acoustic coordinate transformation theory^3^, constitutive parameters for the resultant conventional cloak are obtained from the Jacobin matrix $J = {{\partial (x_{1} ,y_{1} )} \mathord{\left/ {\vphantom {{\partial (x_{1} ,y_{1} )} {\partial (x_{0} ,y_{0} )}}} \right. \kern-\nulldelimiterspace} {\partial (x_{0} ,y_{0} )}}$ as follows:

2 $$ \left\{ {\begin{array}{*{20}ll}    {\overline{\overline{\rho }} _{{cloak}}  = \det (J_{1} )(J_{1} ^{{ - 1}} )^{T} \rho _{0} (J_{1} ^{{ - 1}} ) = \left[ {\begin{array}{*{20}c}    {\rho _{{cloak}}^{{xx}} } & {\rho _{{cloak}}^{{xy}} }  \\    {\rho _{{cloak}}^{{xy}} } & {\rho _{{cloak}}^{{yy}} }  \\   \end{array} } \right] = \rho _{0} \left[ {\begin{array}{*{20}c}    {J_{0}  + m_{{AB}}^{2} J_{0}^{{ - 1}} } & { - m_{{AB}} J_{0}^{{ - 1}} }  \\    { - m_{{AB}} J_{0}^{{ - 1}} } & {J_{0}^{{ - 1}} }  \\   \end{array} } \right]}  \\    {\kappa _{{cloak}}  = \kappa _{0} \det ^{{ - 1}} (J_{1} ) = \begin{array}{*{20}c}    {\kappa _{0} J_{0}^{{ - 1}} } & {\begin{array}{*{20}c}    {\begin{array}{*{20}c}    {} & {} & {\begin{array}{*{20}c}    {} & {\begin{array}{*{20}c}    {\begin{array}{*{20}c}    {\begin{array}{*{20}c}    {} & {}  \\   \end{array} } & {} & {}  \\   \end{array} } & {} & {}  \\   \end{array} }  \\   \end{array} } & {}  \\   \end{array} } & {} & {} & {}  \\   \end{array} } & {}  \\   \end{array} }  \\   \end{array} } \right.$$

wherein $\rho_{0}$ and $\kappa_{0}$ are the mass density and bulk modulus of the host fluid and $J_{0} = {{Y_{C} } \mathord{\left/ {\vphantom {{Y_{C} } {\left( {Y_{C} - Y_{A} } \right)}}} \right. \kern-\nulldelimiterspace} {\left( {Y_{C} - Y_{A} } \right)}}$. For the second step depicted in Fig. 2a,b, the transformation function related with the SSHB region is given by:

3 $$ \Delta KBE \to \Delta KBA:\begin{array}{*{20}c} {} & {\begin{array}{*{20}c} {x_{2} = \left( {\frac{{m_{AB} }}{{m_{AB} - m_{AE} }}} \right)x_{1} + \left( {\frac{{ - m_{AE} m_{KB} }}{{m_{AB} - m_{AE} }}} \right)y_{1} + \left( {\frac{{ - m_{AB} X_{E} }}{{m_{AB} - m_{AE} }}} \right)\,\,\,\,\,\,\,} \\ {y_{2} = \left( {\frac{1}{{m_{AB} - m_{AE} }}} \right)x_{1} + \left( {1 - \frac{{m_{AE} }}{{m_{AB} - m_{AE} }}} \right)y_{1} + \left( {\frac{{ - X_{B} }}{{m_{AB} - m_{AE} }}} \right)} \\ \end{array} } \\ \end{array} $$

The above transformation function folds the $\Delta KBE$ region in the reference space $\left( {x_{1} ,y_{1} } \right)$ to the $\Delta KBA$ region in the real space $\left( {x_{2} ,y_{2} } \right)$ and makes an illusionary SHB on the $\overline{AB}$ boundary as illustrated in Fig. 2c. By employing the corresponding Jacobin matrix, the constitutive parameters of the SSHB region are obtained as:

4 $$ \left\{ {\begin{array}{*{20}ll} {\overline{\overline{\rho }}_{SSHB} = \left[ {\begin{array}{*{20}c} {\rho_{SSHB}^{xx} } & {\rho_{SSHB}^{xy} } \\ {\rho_{SSHB}^{xy} } & {\rho_{SSHB}^{yy} } \\ \end{array} } \right] = \rho_{0} \left[ {\begin{array}{*{20}c} {\frac{{(1 + (m_{BA} - m_{EA} - m_{KB} )^{2} )}}{{m_{AB} (m_{BA} - m_{EA} - m_{KB} ) + m_{AE} m_{AB} }}} & { - \frac{{m_{KB} X_{E} (m_{BA} - m_{EA} - m_{KB} ) + X_{B} }}{{X_{B} (m_{BA} - m_{EA} - m_{KB} ) - X_{E} m_{AB} }}} \\ { - \frac{{m_{KB} X_{E} (m_{BA} - m_{EA} - m_{KB} ) + X_{B} }}{{X_{B} (m_{BA} - m_{EA} - m_{KB} ) - X_{E} m_{AB} }}} & { - \frac{{X_{B}^{2} + m_{KB}^{2} X_{E}^{2} }}{{X_{B} (m_{BA} - m_{EA} - m_{KB} ) - X_{E} m_{AB} }}} \\ \end{array} } \right]} \\ {\kappa_{SSHB} = \kappa_{0} \frac{{(m_{BA} - m_{EA} )^{2} }}{{m_{AB} (m_{BA} - m_{EA} - m_{KB} ) + m_{AE} m_{AB} }}\begin{array}{*{20}c} {\begin{array}{*{20}c} {\begin{array}{*{20}c} {\begin{array}{*{20}c} {\begin{array}{*{20}c} {\begin{array}{*{20}c} {} & {} \\ \end{array} } & {\begin{array}{*{20}c} {\begin{array}{*{20}c} {\begin{array}{*{20}c} {\begin{array}{*{20}c} {} & {} \\ \end{array} } & {} \\ \end{array} } & {} \\ \end{array} } & {} & {} \\ \end{array} } \\ \end{array} } & {} \\ \end{array} } & {} & {} \\ \end{array} } & {} & {} & {} \\ \end{array} } & {} & {} & {} \\ \end{array} } \\ \end{array} } \right. $$

Then, corresponding to the third step illustrated in Fig. 4a,b, the transformation equation that maps the carpet cloak with SSHBs in the reference space $\left( {x_{2} ,y_{2} } \right)$ to non-closed compressed regions denoted by region 1 in the real space $\left( {x_{3} ,y_{3} } \right)$ is expressed as follows:

5 $$ \Delta OCB \to \Delta OC^{\prime}B:\begin{array}{*{20}c} {} & {\begin{array}{*{20}c} {x_{3} = x_{2} + km_{{OC^{\prime}}} y_{2} } \\ {y_{3} = ky_{2} \,\,\,\,\,\,\,\,\,\,\,\,\,\,\,\,\,\,\,} \\ \end{array} } \\ \end{array} $$

where $k = \frac{{Y_{C^{\prime}} }}{{Y_{C} }}$, represents the compressing ratio. The above transformation function gives the material properties of each different part of region 1 (Fig. 4b) as:

6 $$ \left\{ {\begin{array}{*{20}ll} \begin{gathered} \overline{\overline{\rho }}_{BA^{\prime}C^{\prime}} = \left[ {\begin{array}{*{20}c} {k\rho_{cloak}^{xx} } & {\rho_{cloak}^{xy} - km_{{OC^{\prime}}} \rho_{cloak}^{xx} } \\ {\rho_{cloak}^{xy} - km_{OC} \rho_{cloak}^{xx} } & {\begin{array}{*{20}c} {} & {} \\ \end{array} km_{{OC^{\prime}}} \rho_{cloak}^{xx} + k^{ - 1} \rho_{cloak}^{yy} - 2m_{{OC^{\prime}}} \rho_{cloak}^{xy} } \\ \end{array} } \right],\kappa_{BA^{\prime}C^{\prime}} = \frac{1}{k}\kappa_{cloak} \begin{array}{*{20}c} {} & {} \\ \end{array} \hfill \\ \hfill \\ \end{gathered} \\ \begin{gathered} \overline{\overline{\rho }}_{BK^{\prime}A^{\prime}} = \left[ {\begin{array}{*{20}c} {k\rho_{SSHB}^{xx} } & {\rho_{SSHB}^{xy} - km_{{OC^{\prime}}} \rho_{SSHB}^{xx} } \\ {\rho_{SSHB}^{xy} - km_{{OC^{\prime}}} \rho_{SSHB}^{xx} } & {\begin{array}{*{20}c} {} & {} \\ \end{array} km_{{OC^{\prime}}} \rho_{SSHB}^{xx} + k^{ - 1} \rho_{SSHB}^{yy} - 2m_{{OC^{\prime}}} \rho_{SSHB}^{xy} } \\ \end{array} } \right],\kappa_{BK^{\prime}A^{\prime}} = \frac{1}{k}\kappa_{SSHB} \hfill \\ \hfill \\ \end{gathered} \\ {\overline{\overline{\rho }}_{BEK^{\prime}} = \rho_{0} \left[ {\begin{array}{*{20}c} k & { - km_{OC^{\prime}} } \\ { - km_{OC^{\prime}} } & {km_{OC^{\prime}}^{2} + k^{ - 1} } \\ \end{array} } \right],\kappa_{BEK^{\prime}} = \frac{1}{k}\kappa_{0} \begin{array}{*{20}c} {\begin{array}{*{20}c} {} & {\begin{array}{*{20}c} {\begin{array}{*{20}c} {\begin{array}{*{20}c} {\begin{array}{*{20}c} {} & {} \\ \end{array} } & {} \\ \end{array} } & {} & {} & {} \\ \end{array} } & {} & {} & {} \\ \end{array} } & {} & {} \\ \end{array} } & {} \\ \end{array} } \\ \end{array} } \right. $$

Similarly, the compression transformation functions are applied to the surrounding host fluid in order to achieve region 2 illustrated in Fig. 4b. The $\Delta BMC$ in the reference space $\left( {x_{2} ,y_{2} } \right)$ is compressed to $\Delta BMC^{\prime}$ in the real space $\left( {x_{3} ,y_{3} } \right)$ with the transformation function:

7 $$ \Delta BMC \to \Delta BMC^{\prime}:\begin{array}{*{20}c} {} &{\begin{array}{*{20}c} \begin{gathered} x_{3} = \underbrace {{\left( {\frac{{X_{B} Y_{M} - X_{B} Y_{C} + X_{M} Y_{C} - X_{C^{\prime}} Y_{M} }}{{\Delta_{0} }}} \right)}}_{{a_{0} }}x_{2} + \underbrace {{\left( {\frac{{X_{M} X_{C^{\prime}} - X_{B} X_{C^{\prime}} }}{{\Delta_{0} }}} \right)}}_{{b_{0} }}y_{2} \begin{array}{*{20}c} {\begin{array}{*{20}c} {\begin{array}{*{20}c} {\begin{array}{*{20}c} {} &{} \\ \end{array} } &{\begin{array}{*{20}c} {} \\ \end{array} } \\ \end{array} } &{} \\ \end{array} } &{} \\ \end{array} \hfill \\ \begin{array}{*{20}c} {} &{} \\ \end{array} \quad + \underbrace {{\left( {\frac{{X_{B} X_{C^{\prime}} Y_{M} - X_{B} X_{C} Y_{M} }}{{\Delta_{0} }}} \right)}}_{{c_{0} }} \hfill \\ \end{gathered} \\ {y_{3} = \underbrace {{\left( {\frac{{Y_{M} Y_{C} - Y_{M} Y_{C^{\prime}} }}{{\Delta_{0} }}} \right)}}_{{d_{0} }}x_{2} + \underbrace {{\left( {\frac{{X_{B} Y_{M} - X_{B} Y_{C^{\prime}} + X_{M} Y_{C^{\prime}} }}{{\Delta_{0} }}} \right)}}_{{e_{0} }}y_{2} + \underbrace {{\left( {\frac{{X_{B} Y_{M} Y_{C^{\prime}} - X_{B} Y_{M} Y_{C} }}{{\Delta_{0} }}} \right)}}_{{f_{0} }}} \\ \end{array} } \\ \end{array} $$

wherein $\Delta_{0} = X_{B} Y_{M} - X_{B} Y_{C} + X_{M} Y_{C} - X_{C} Y_{M}$. In a similar manner, $\vartriangle ENA$ in the reference space $\left( {x_{2} ,y_{2} } \right)$ is also compressed to $\vartriangle ENA^{\prime}$ in the real space $\left( {x_{3} ,y_{3} } \right)$ by following coordinate transformation:

8 $$ \Delta ENA \to \Delta ENA^{\prime}:\begin{array}{*{20}c} {} & {\begin{array}{*{20}c} {x_{3} = \underbrace {{\left( {\frac{{ - X_{E} Y_{A} + X_{N} Y_{A} - X_{A^{\prime}} Y_{N} + X_{E} Y_{N} }}{{\Delta_{1} }}} \right)}}_{{a_{1} }}x_{2} + \underbrace {{\left( {\frac{{X_{N} X_{A^{\prime}} - X_{E} X_{A^{\prime}} }}{{\Delta_{1} }}} \right)}}_{{b_{1} }}y_{2} + \underbrace {{\left( {\frac{{X_{E} X_{A^{\prime}} Y_{N} }}{{\Delta_{1} }}} \right)}}_{{c_{1} }}} \\ {y_{3} = \underbrace {{\left( {\frac{{Y_{N} Y_{A^{\prime}} - Y_{N} Y_{A} }}{{\Delta_{1} }}} \right)}}_{{d_{1} }}x_{2} + \underbrace {{\left( {\frac{{X_{N} Y_{A^{\prime}} - X_{E} Y_{A^{\prime}} + X_{E} Y_{N} }}{{\Delta_{1} }}} \right)}}_{{e_{1} }}y_{2} + \underbrace {{\left( {\frac{{X_{E} Y_{A^{\prime}} Y_{N} - X_{E} Y_{A} Y_{N} }}{{\Delta_{1} }}} \right)}}_{{f_{1} }}\,\,\,\,\,\,\,} \\ \end{array} } \\ \end{array} $$

that $\Delta_{1} = X_{N} Y_{A} - X_{E} Y_{A} + X_{E} Y_{N} - X_{N} Y_{E}$. According to Eqs. (7) and (8), the constitutive materials of $\vartriangle ENA^{\prime}$ and $\vartriangle BMC^{\prime}$ that construct region 2 in Fig. 4b are expressed as:

9 $$ \left\{ {\begin{array}{*{20}c} {\overline{\overline{\rho }}_{BMC^{\prime}} = \rho_{0} \left[ {\begin{array}{*{20}c} {\frac{{d_{0}^{2} + e_{0}^{2} }}{{a_{0} e_{0} - b_{0} d_{0} }}} & {\frac{{a_{0} d_{0} - b_{0} e_{0} }}{{a_{0} e_{0} - b_{0} d_{0} }}} \\ {\frac{{a_{0} d_{0} - b_{0} e_{0} }}{{a_{0} e_{0} - b_{0} d_{0} }}} & {\frac{{a_{0}^{2} + b_{0}^{2} }}{{a_{0} e_{0} - b_{0} d_{0} }}} \\ \end{array} } \right],\kappa_{BMC^{\prime}} = \frac{1}{{a_{0} e_{0} - b_{0} d_{0} }}\kappa_{0} } \\ {\overline{\overline{\rho }}_{ENA^{\prime}} = \rho_{0} \left[ {\begin{array}{*{20}c} {\frac{{d_{1}^{2} + e_{1}^{2} }}{{a_{1} e_{1} - b_{1} d_{1} }}} & {\frac{{a_{1} d_{1} - b_{1} e_{1} }}{{a_{1} e_{1} - b_{1} d_{1} }}} \\ {\frac{{a_{1} d_{1} - b_{1} e_{1} }}{{a_{1} e_{1} - b_{1} d_{1} }}} & {\frac{{a_{1}^{2} + b_{1}^{2} }}{{a_{1} e_{1} - b_{1} d_{1} }}} \\ \end{array} } \right],\kappa_{ENA^{\prime}} = \frac{1}{{a_{1} e_{1} - b_{1} d_{1} }}\kappa_{0} \begin{array}{*{20}c} {} & {} \\ \end{array} } \\ \end{array} } \right. $$

Subsequently, the complementary medium $GHA^{\prime}C^{\prime}$ denoted by region 3 in Fig. 4b is derived by applying the transformation function:

10 $$ \Delta OGC \to \Delta OGC^{\prime}:\begin{array}{*{20}c} {} & {\begin{array}{*{20}c} {x_{3} = \left( {1 - k\frac{{m_{{OC^{\prime}}} }}{{m_{OG} }}} \right)x_{2} + km_{{OC^{\prime}}} y_{2} } \\ {y_{3} = \left( {\frac{1}{{m_{OG} }}\left( {1 - k} \right)} \right)x_{2} + km_{{OC^{\prime}}} y_{2} } \\ \end{array} } \\ \end{array} $$

which folds the $\overline{GH}$ boundary to itself and $\overline{AC}$ to $\overline{A^{\prime}C^{\prime}}$. Equation (10) gives the material properties of the polygon region $GHC^{\prime}A^{\prime}$ denoted by region 3 in Fig. 4b as follows:

11 $$ \left\{ {\begin{array}{*{20}ll} {\overline{\overline{\rho }}_{GHC^{\prime}A^{\prime}} = \rho_{0} \left[ {\begin{array}{*{20}c} {\frac{{ - k^{2} m_{OC^{\prime}}^{2} m_{OG}^{2} - (k - 1)^{2} }}{{km_{OC^{\prime}} m_{OG} (1 - k + km_{OC^{\prime}} - m_{OG} )}}} & {\frac{{k^{2} m_{OC^{\prime}}^{2} m_{OG}^{2} - (k - 1)(m_{OG} - km_{OC^{\prime}} )}}{{km_{OC^{\prime}} m_{OG} (1 - k + km_{OC^{\prime}} - m_{OG} )}}} \\ {\frac{{k^{2} m_{OC^{\prime}}^{2} m_{OG}^{2} - (k - 1)(m_{OG} - km_{OC^{\prime}} )}}{{km_{OC^{\prime}} m_{OG} (1 - k + km_{OC^{\prime}} - m_{OG} )}}} & {\frac{{ - k^{2} m_{OC^{\prime}}^{2} m_{OG}^{2} - (m_{OG} - km_{OC^{\prime}} )^{2} }}{{km_{OC^{\prime}} m_{OG} (1 - k + km_{OC^{\prime}} - m_{OG} )}}} \\ \end{array} } \right]} \\ {\kappa_{GHC^{\prime}A^{\prime}} = \begin{array}{*{20}c} {\begin{array}{*{20}c} {\begin{array}{*{20}c} {\frac{{ - \kappa_{0} m_{OG} }}{{km_{OC^{\prime}} m_{OG} (1 - k + km_{OC^{\prime}} - m_{OG} )}}} & {\begin{array}{*{20}c} {\begin{array}{*{20}c} {\begin{array}{*{20}c} {\begin{array}{*{20}c} {} & {} \\ \end{array} } & {} & {} & {} \\ \end{array} } & {} & {} & {} \\ \end{array} } & {} & {} & {} \\ \end{array} } \\ \end{array} } & {} \\ \end{array} } & {} \\ \end{array} } \\ \end{array} } \right. $$

Finally, transformation equations are applied to the specify region 4 in Fig. 4b. The $\Delta GMC^{\prime}$ domain of region 4 in the real space $\left( {x_{3} ,y_{3} } \right)$ is determined by the transformation function:

12 $$ \Delta GMC \to \Delta GMC^{\prime}:\begin{array}{*{20}c} {} &{\begin{array}{*{20}c} \begin{gathered} x_{3} = \underbrace {{\left( {\frac{{X_{M} Y_{C} - X_{{C^{\prime}}} Y_{M} - X_{M} Y_{G} + X_{G} Y_{M} - X_{G} Y_{C} + X_{{C^{\prime}}} Y_{G} }}{{\Delta_{2} }}} \right)}}_{{a_{2} }}x_{2} +\underbrace {{\left( {\frac{{X_{M} X_{{C^{\prime}}} - X_{{C^{\prime}}} X_{G} }}{{\Delta_{2} }}} \right)}}_{{b_{2} }}y_{2} \hfill \\ \quad \begin{array}{*{20}c} {} &{} \\ \end{array} +\quad  \underbrace {{\left( {\frac{{X_{{C^{\prime}}} X_{G} Y_{M} - X_{M} X_{{C^{\prime}}} Y_{G} }}{{\Delta_{2} }}} \right)}}_{{c_{2} }} \hfill \\ \hfill \\ \end{gathered} \\ \begin{gathered} y_{3} = \underbrace {{\left( {\frac{{Y_{M} Y_{C} - Y_{M} Y_{{C^{\prime}}} - Y_{C} Y_{G} - Y_{{C^{\prime}}} Y_{G} }}{{\Delta_{2} }}} \right)}}_{{d_{2} }}x_{2} + \underbrace {{\left( {\frac{{X_{M} Y_{{C^{\prime}}} - X_{M} Y_{G} + X_{G} Y_{M} - X_{G} Y_{{C^{\prime}}} }}{{\Delta_{2} }}} \right)}}_{{e_{2} }}y_{2} \begin{array}{*{20}c} {} &{} \\ \end{array} \hfill \\\quad  \begin{array}{*{20}c} {} &{} \\ \end{array} + \quad  \underbrace {{\left( {\frac{{X_{M} Y_{C} Y_{G} - X_{G} Y_{M} Y_{C} - X_{M} Y_{{C^{\prime}}} Y_{G} + X_{G} Y_{M} Y_{{C^{\prime}}} }}{{\Delta_{2} }}} \right)}}_{{f_{2} }} \hfill \\ \end{gathered} \\ \end{array} } \\ \end{array} $$

wherein $\Delta_{3} = X_{H} Y_{A} - X_{N} Y_{A} + X_{N} Y_{H} - X_{H} Y_{N}$. The coordinate transformation described in Eq. (12) folds the $\overline{MC}$ boundary in the reference space $\left( {x_{2} ,y_{2} } \right)$ to $\overline{MC^{\prime}}$ in the real space $\left( {x_{3} ,y_{3} } \right)$ and also folds the outer boundary $\overline{MG}$ to itself. Moreover, the $\Delta HNA^{\prime}$ domain of region 4 in the real space $\left( {x_{3} ,y_{3} } \right)$ is determined by the transformation equation:

13 $$ \Delta HNA \to \Delta HNA^{\prime}:\begin{array}{*{20}c} {} & {\begin{array}{*{20}c} \begin{gathered} x_{3} = \underbrace {{\left( {\frac{{ - X_{N} Y_{A} + X_{{A^{\prime}}} Y_{N} + X_{H} Y_{A} - X_{{A^{\prime}}} Y_{H} + X_{N} Y_{H} - X_{H} Y_{N} }}{{\Delta_{3} }}} \right)}}_{{a_{3} }}x_{2} + \underbrace {{\left( {\frac{{X_{H} X_{{A^{\prime}}} - X_{{A^{\prime}}} X_{N} }}{{\Delta_{3} }}} \right)}}_{{b_{3} }}y_{2} \hfill \\ \quad \begin{array}{*{20}c} {} & {} \\ \end{array} + \underbrace {{\left( {\frac{{X_{{A^{\prime}}} X_{N} Y_{A} - X_{{A^{\prime}}} X_{H} Y_{N} }}{{\Delta_{3} }}} \right)}}_{{c_{3} }} \hfill \\ \hfill \\ \end{gathered} \\ \begin{gathered} y_{3} = \underbrace {{\left( {\frac{{ - Y_{A} Y_{N} + Y_{{A^{\prime}}} Y_{N} + Y_{A} Y_{H} - Y_{{A^{\prime}}} Y_{H} }}{{\Delta_{3} }}} \right)}}_{{d_{3} }}x_{2} + \underbrace {{\left( {\frac{{ - X_{N} Y_{{A^{\prime}}} + X_{H} Y_{{A^{\prime}}} + X_{H} Y_{N} - X_{H} Y_{N} }}{{\Delta_{3} }}} \right)}}_{{e_{3} }}y_{2} \begin{array}{*{20}c} {} & {} \\ \end{array} \hfill \\ \quad \begin{array}{*{20}c} {} & {} \\ \end{array} + \underbrace {{\left( {\frac{{X_{H} Y_{A} Y_{N} - X_{N} Y_{A} Y_{H} + X_{N} Y_{{A^{\prime}}} Y_{A} - X_{H} Y_{N} Y_{{A^{\prime}}} }}{{\Delta_{3} }}} \right)}}_{{f_{3} }} \hfill \\ \end{gathered} \\ \end{array} } \\ \end{array} $$

with the assumption of $\Delta_{2} = X_{M} Y_{C} - X_{M} Y_{G} + X_{G} Y_{M} - X_{G} Y_{C}$. The coordinate transformation presented in Eq. (13) folds the $\overline{NA}$ boundary in reference space $\left( {x_{2} ,y_{2} } \right)$ to $\overline{NA^{\prime}}$ in the real space $\left( {x_{3} ,y_{3} } \right)$ and folds the outer boundary $\overline{HN}$ to itself. Equations (12) and (13), respectively give the material properties of $\vartriangle GMC^{\prime}$ and $\vartriangle HNA^{\prime}$ domains of region 4 as follows:

14 $$ \left\{ {\begin{array}{*{20}c} {\overline{\overline{\rho }}_{{GMC^{\prime}}} = \rho_{0} \left[ {\begin{array}{*{20}c} {\frac{{d_{2}^{2} + e_{2}^{2} }}{{a_{2} e_{2} - b_{2} d_{2} }}} & {\frac{{a_{2} d_{2} - b_{2} e_{2} }}{{a_{2} e_{2} - b_{2} d_{2} }}} \\ {\frac{{a_{2} d_{2} - b_{2} e_{2} }}{{a_{2} e_{2} - b_{2} d_{2} }}} & {\frac{{a_{2}^{2} + b_{2}^{2} }}{{a_{2} e_{2} - b_{2} d_{2} }}} \\ \end{array} } \right],\;\kappa_{{GMC^{\prime}}} = \frac{1}{{a_{2} e_{2} - b_{2} d_{2} }}\kappa_{0} } \\ {\overline{\overline{\rho }}_{{HNA^{\prime}}} = \rho_{0} \left[ {\begin{array}{*{20}c} {\frac{{d_{3}^{2} + e_{3}^{2} }}{{a_{3} e_{3} - b_{3} d_{3} }}} & {\frac{{a_{3} d_{3} - b_{3} e_{3} }}{{a_{3} e_{3} - b_{3} d_{3} }}} \\ {\frac{{a_{3} d_{3} - b_{3} e_{3} }}{{a_{3} e_{3} - b_{3} d_{3} }}} & {\frac{{a_{3}^{2} + b_{3}^{2} }}{{a_{3} e_{3} - b_{3} d_{3} }}} \\ \end{array} } \right],\;\kappa_{{HNA^{\prime}}} = \frac{1}{{a_{3} e_{3} - b_{3} d_{3} }}\kappa_{0} } \\ \end{array} } \right. $$

The presented three design steps illustrated in Figs. 2 and 4 to achieve a non-closed carpet cloak are demonstrated by mathematical terminology (Eqs. 1–14). As illustrated in the next section, all discussions can be repeated to designing the non-closed unidirectional cloak because of its similar design steps with the carpet cloak. The design method also can be extended to achieve a larger number of windows in the structure and it could also be applied to other acoustic devices to make them fenestrated. The method could also be generalized to illusion devices by changing the first reference space in the step 1 (Fig. 2).

### Non-closed unidirectional cloak

**Figure 6:**
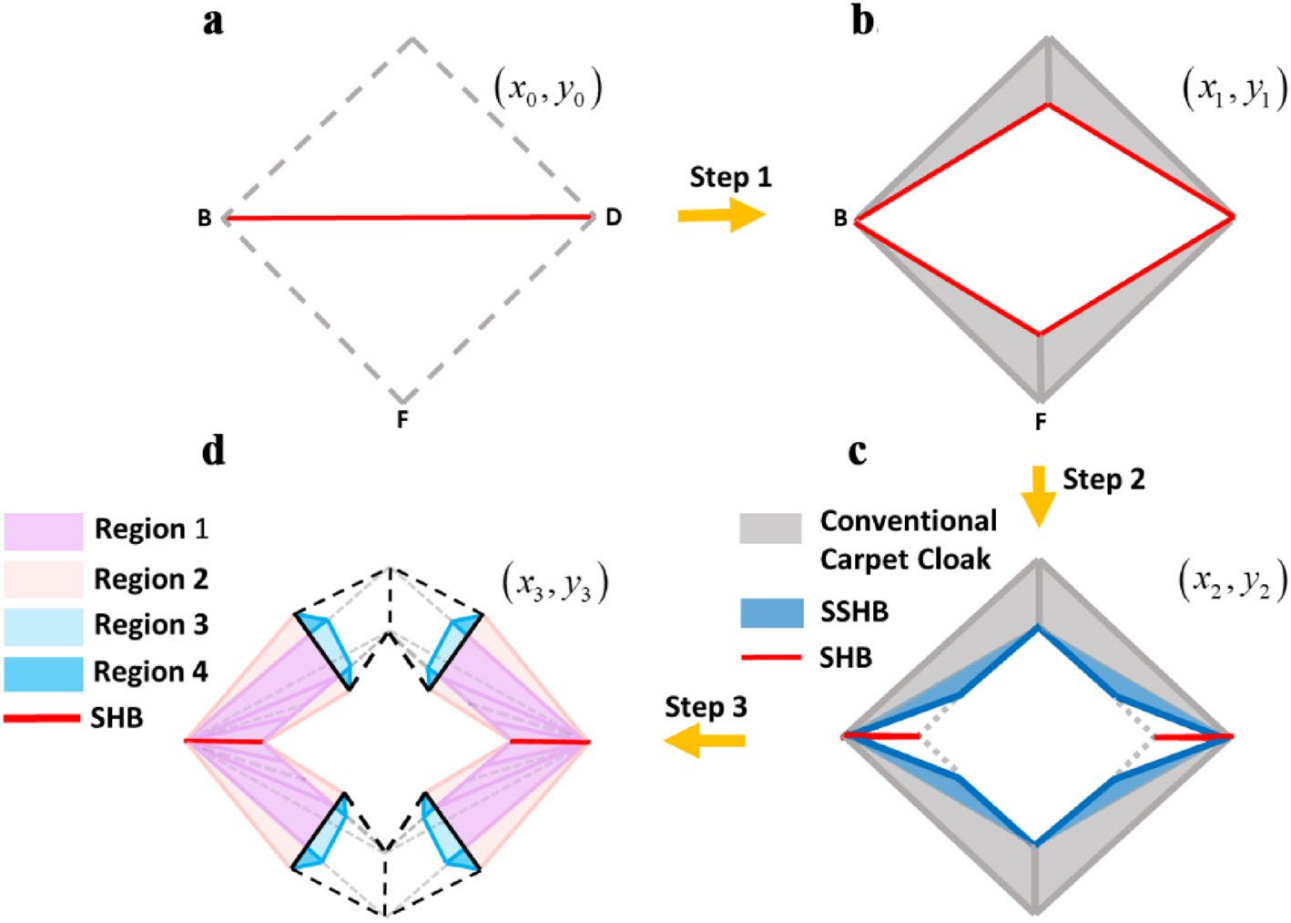
Scheme illustration of the free space fenestrated cloak (**a**–**d**) The three design steps. The unidirectional cloak is achieved by transforming the diamond space specified by grey dashed line to the space with the diamond shape inner sound hard boundaries. Analogous to the non-closed carpet cloak scenario, firstly one should use SSHB material for the unidirectional cloak rather than sound hard boundary condition. (**d**) Geometry of resulting device. The proposed structure can hide objects located in the host fluid without making them blinded and the drawn windows of structure, allow transforming of matter and information.

It is well understood that carpet cloaks only work in the presence of a reflecting plane. In order to make it possible to hide an arbitrary object in the free space, the proposed method is further extended to design a non-closed unidirectional cloak. Analogous to the EM scenario^21^, designing acoustic unidirectional cloaks is based on the fact that there is no scattered field when the propagation of acoustic wave is parallel to an infinitely thin SHB surface. Figure 6a,b illustrate the idea of unidirectional cloak. As illustrated, to design such a unidirectional cloak, an SHB diamond hole is made in $BCDF$ domain. For detail, the reference space is compressed into the real space by mapping an SHB line segment to an SHB diamond in the interior boundary, while the exterior space is unchanged. Any object located inside the resultant diamond shape SHB region becomes invisible. Unidirectional free space cloaks as a practical and easy to fabricate alternative device for ideal free space cloaks have numerous particular applications such as hiding submarines from sonar, airborne sound cloaking, etc.

In order to make the conventional unidirectional cloak non-closed to outer world, the aforementioned three design steps, i.e. transforming the structure to the device with SSHBs, compressing and complementing, should be employed to make windows on the body of it. Due to the structural symmetry of the unidirectional cloak and similar materials with the carpet cloak, the reference space can be considered as two mirror conventional carpet cloaks, as illustrated in Fig. 6b. Hence, by passing the same three design steps, the resultant transformation media will have the similar constitutive materials with the non-closed carpet cloak. In Fig. 6, second and third design steps are applied to achieve a non-closed unidirectional cloak.

**Figure 7:**
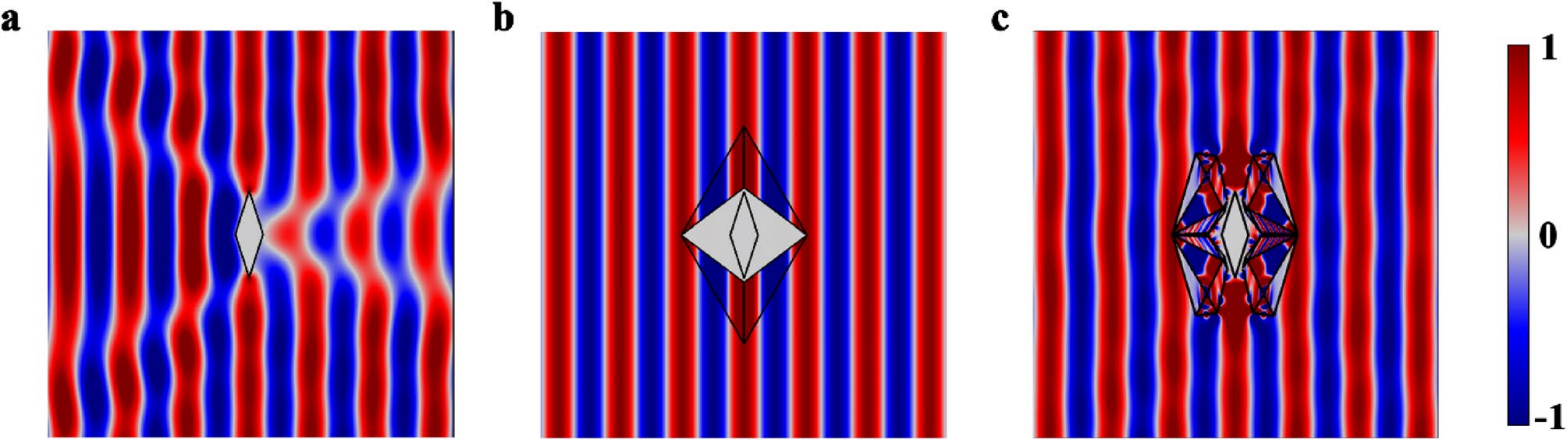
The simulation results of non-closed free space cloak. The near field of (**a**) object with sound hard boundaries, (**b**) the conventional unidirectional cloak, and (**c**) the target in the presence of non-closed free space cloak, which is recovered as well as the conventional free space cloaks pattern in (**b**).

By performing numerical simulations, the directional perfect cloaking effect of the resultant non-closed free space cloak is verified. Figure 7a–c demonstrate the near field distribution of three cases: an arbitrary object in free space, conventional and non-closed unidirectional cloaks. The incident plane wave is excited parallel to the virtual SHB surface. Comparing the results of Fig. 7b,c underlines the closely equivalent signature of the non-closed cloak and original one.

Consequently, the performance of free space cloak with the advantage of the non-closed structure is verified. The important prudence is to note that the non-closed free space cloak is prescribed for a specific directional propagation similar to the conventional one. The proposed non-closed unidirectional cloak possesses all excellences of the conventional unidirectional cloak, including the object independent performance and homogeneous-material structure, together with the non-blinded architecture. All these interesting benefits make the device applicable in free space cloaking practical demands.

## Meta-atom realization

In this section, we propose a possible acoustic metamaterial structure that could be utilized to mimic the constitutive parameters of the NCAC structure. To this end, considering the effective medium theory^44–48^, we design meta-atoms whose effective properties are matched with the required data presented in Table 1. At the first, the off-diagonal components of the mass density tensor should be eliminated. It is worth mention that any off-diagonal symmetric tensor can be transformed to a diagonal one with a proper rotation around its principal axis^49^. To this aim, the obtained tensors presented in Table. 1 will be multiplied by the rotation matrix ($\theta$ is unknown and must be calculated).

15 $$ \left[ {\begin{array}{*{20}c} {\rho_{u} } & 0 \\ 0 & {\rho_{v} } \\ \end{array} } \right] = \left[ {\begin{array}{*{20}c} {\cos \theta } & { - \sin \theta } \\ {\sin \theta } & {\cos \theta } \\ \end{array} } \right] \times \left[ {\begin{array}{*{20}c} {\rho_{xx} } & {\rho_{xy} } \\ {\rho_{xy} } & {\rho_{yy} } \\ \end{array} } \right] \times \left[ {\begin{array}{*{20}c} {\cos \theta } & {\sin \theta } \\ { - \sin \theta } & {\cos \theta } \\ \end{array} } \right] = \left[ {\begin{array}{*{20}c} {t_{11} } & {t_{12} } \\ {t_{21} } & {t_{22} } \\ \end{array} } \right] $$

where

16 $$ t_{12} = t_{21} = \left( {\frac{{\rho_{xx} - \rho_{yy} }}{2}} \right)\sin 2\theta + \rho_{xy} \cos 2\theta $$

In Eq. (15) v and u denote the axes of the rotated coordinate. In order to find the rotation angle which will cause the tensor of Eq. (15) to be diagonal, we should solve $t_{12} = t_{21} = 0$ which consequently results in $\theta = \frac{1}{2}\arctan \left( {\frac{{ - 2\rho_{xy} }}{{\rho_{xx} - \rho_{yy} }}} \right)$ and the components of anisotropic mass density in new coordinate, can be calculated as

17 $$ \left\{ {\begin{array}{*{20}c} {\rho_{u} = \cos^{2} \theta \rho_{xx} + \sin^{2} \theta \rho_{yy} + 2\sin \theta \cos \theta \rho_{xy} } \\ {\rho_{v} = \sin^{2} \theta \rho_{xx} + \cos^{2} \theta \rho_{yy} - 2\sin \theta \cos \theta \rho_{xy} } \\ \end{array} } \right. $$

By applying aforementioned coordinate rotation, the diagonalized constitutive parameters are presented in Table 2.

**Table 2:** The constitutive materials of the NCAC's regions after diagonalization.

Region 1	$$\theta^{rot} = - 28.06^{ \circ } ,\overline{\overline{\rho }}_{BA^{\prime}C^{\prime}} = \rho_{0} \left[ {\begin{array}{*{20}c} {{0}{\text{.3916}}} & {0} \\ {0} & {{2}{\text{.5539}}} \\ \end{array} } \right],\kappa_{BA^{\prime}C^{\prime}} = {2}{\text{.8441}}\kappa_{0}$$
	$$\theta^{rot} = 14.44^{ \circ } ,\overline{\overline{\rho }}_{BK^{\prime}E} = \rho_{0} \left[ {\begin{array}{*{20}c} {{0}{\text{.6128}}} & {0} \\ {0} & {{1}{\text{.6319}}} \\ \end{array} } \right],\kappa_{BK^{\prime}E} = {1}{\text{.4789}}\kappa_{0}$$
	$$\theta^{rot} = 40.26^{ \circ } ,\overline{\overline{\rho }}_{BA^{\prime}K^{\prime}} = \rho_{0} \left[ {\begin{array}{*{20}c} {{ - 18}{\text{.557}}} & {0} \\ 0 & {{ - 0}{\text{.0539}}} \\ \end{array} } \right],\kappa_{BA^{\prime}K^{\prime}} = { - 0}{\text{.1056}}\kappa_{0}$$
Region 2	$$\theta^{rot} = 0.51^{ \circ } ,\overline{\overline{\rho }}_{BMC^{\prime}} = \rho_{0} \left[ {\begin{array}{*{20}c} {{3}{\text{.6443}}} & 0 \\ 0 & {{0}{\text{.2744}}} \\ \end{array} } \right],\kappa_{BMC^{\prime}} = {1}{\text{.4789}}\kappa_{0}$$
	$$\theta^{rot} = - 7.41^{ \circ } ,\overline{\overline{\rho }}_{ENA^{\prime}} = \rho_{0} \left[ {\begin{array}{*{20}c} {{0}{\text{.3941}}} & 0 \\ 0 & {{2}{\text{.5372}}} \\ \end{array} } \right],\kappa_{ENA^{\prime}} = {1}{\text{.4789}}\kappa_{0}$$
Region 3	$$\theta^{rot} = 8.5^{ \circ } ,\overline{\overline{\rho }}_{GHC^{\prime}A^{\prime}} = \rho_{0} \left[ {\begin{array}{*{20}c} {{ - 5}{\text{.4}}} & {0} \\ {0} & {{ - 0}{\text{.18}}} \\ \end{array} } \right],\kappa_{GHC^{\prime}A^{\prime}} = { - 1}{\text{.3897}}\kappa_{0}$$
Region 4	$$\theta^{rot} = - 34.65^{ \circ } ,\overline{\overline{\rho }}_{MC^{\prime}G} = \rho_{0} \left[ {\begin{array}{*{20}c} {{ - 1}{\text{.46}}} & 0 \\ 0 & { - 0.68} \\ \end{array} } \right],\kappa_{MC^{\prime}G} = { - 1}{\text{.3897}}\kappa_{0}$$
	$$\theta^{rot} = 13.77^{ \circ } ,\overline{\overline{\rho }}_{NA^{\prime}H} = \rho_{0} \left[ {\begin{array}{*{20}c} {{ - 8}{\text{.11}}} & 0 \\ 0 & {{ - 0}{\text{.12}}} \\ \end{array} } \right],\kappa_{NA^{\prime}H} = { - 1}{\text{.3897}}\kappa_{0}$$

**Figure 8:**
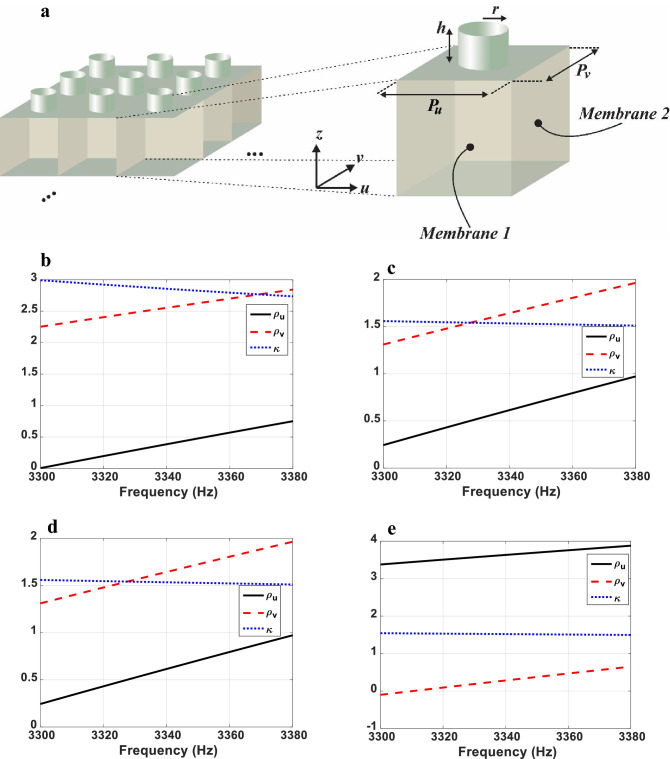

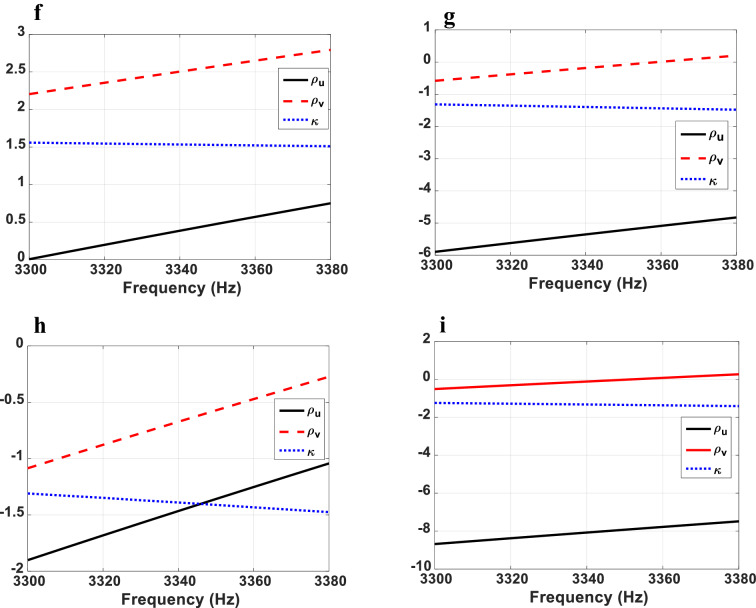
(**a**) Scheme illustration of the utilized non-resonant meta-atom. The membrane in repeated along u and v directions with periodicity $p_{u} = p_{v} = 4\,\,mm$. The retrieved parameters for all regions are shown in (**b**–**i**). (**b**–**d**) Real parts of retrieved parameters of region 1. For $\Delta BA^{\prime}C^{\prime}$, the thickness of membranes’ faces are $th_{u} = 6.368\,\,\mu m$ and $th_{v} = 5.387\,\,\mu m$. The radius and height of the side branch are $r = 0.4\,\,mm$ and $h = 2.958\,\,mm$, respectively. For $\Delta BK^{\prime}E$: $th_{u} = 5.859\,\,\mu m$, $th_{v} = 6\,\,\mu m$, $r = 0.4\,\,mm$ and $h = 6.11\,\,mm$. For $\Delta BA^{\prime}K^{\prime}$, $th_{u} = 10.5\,\,\mu m$, $th_{v} = 6.53\,\,\mu m$, $r = 1.2\,\,mm$ and $h = 1\,\,mm$. (**e**,**f**) Real parts of retrieved parameters of region 2. For $\Delta BMC^{\prime}$: $th_{u} = 4.603\,\,\mu m$, $th_{v} = 6.41\,\,\mu m$, $r = 0.4\,\,mm$ and $h = 6.11\,\,mm$ and for $\Delta ENA^{\prime}$, $th_{u} = 6.36\,\,\mu m$, $th_{v} = 5.4\,\,\mu m$, $r = 0.4\,\,mm$ and $h = 6.11\,\,mm$. (**g**) Real parts of retrieved parameters of region 3. For $GHC^{\prime}A^{\prime}$ region, $th_{u} = 8.02\,\,\mu m$, $th_{v} = 6.57\,\,\mu m$, $r = 0.4\,\,mm$ and $h = 0.94\,\,mm$. (**h**,**i**) Real parts of retrieved parameters of region 4. For $\Delta MC^{\prime}G$: $th_{u} = 6.986\,\,\mu m$, $th_{v} = 6.471\,\,\mu m$, $r = 0.4\,\,mm$ and $h = 0.94\,\,mm$ and for $\Delta NA^{\prime}H$, $th_{u} = 8.611\,\,\mu m$, $th_{v} = 6.553\,\,\mu m$, $r = 0.4\,\,mm$ and $h = 0.94\,\,mm$.

As can be seen in Table 2, the required parameters for regions 3 and 4 and $\vartriangle BA^{\prime}K^{\prime}$ region, are double negative value for mass density tensor and also have negative bulk modulus. A prevalent way to implement homogeneous double-negative acoustic media is to use resonant membrane type meta-atoms. However, the inevitable loss and dispersion of the resonant structures, extremely limits the efficiency and operating frequency band of the homogenized medium and exacerbates the unwanted couplings. To go beyond these restrictions, we utilize the quasi two-dimensional non-resonant meta-atom proposed in^48^. As shown in Fig. 8a, the meta-atom consists of an elastic membrane and a side branch with open end. By assigning different thicknesses to membranes’ faces in u and v directions ($th_{u}$ and $th_{v}$, respectively), anisotropic and negative density can be achieved simultaneously. In addition, the bulk modulus is tuned by the geometry of the side branch. The wide bandwidth of this meta-atom is resulted from its large resonance damping. The membrane material is assumed to be aluminum with Young modulus of 70 GPa, the Poisson ratio of 0.33 and the mass density of 2700 kg/m^3^ and the tension on the membrane is assumed to be zero. The dimensions and geometrical features of the meta-atom are described in the Figure caption and the retrieved parameters for all regions are shown in Fig. 8b–i. It can be seen that the retrieved results match well with the required parameters at the frequency of $3.34\,\,kHz$. Due to the non-resonant properties of the meta-atom, the imaginary parts of the retrieved parameters are negligible. It is obvious that the meta-atom exhibits negative mass density and bulk modulus in a broadband frequency range without relying on the resonance and the retrieved parameters, change smoothly with respect to the frequency which minimizes the coupling effects. These results, therefore demonstrate the possibility of realization of the designed non-closed acoustic cloaking devices. The finite element analysis software COMSOL is used for the parameter retrieval simulations.

## Discussion and conclusion

To summarize, we design and numerically demonstrate a new strategy to achieve acoustic external cloaking without full enclosure by applying sequential-step linear coordinate transformations. The cloaking effect of the proposed non-closed devices is independent of the shape and constitutive material of the target. Therefore, the target can alter shape or move in the hidden region and transform information with outer world without being blinded. There is a tradeoff when the window(s) created on the structure are extended, which leads to increasing the value of negative constitutive parameters. The presented approach surmounts resorting to spatially-varying constitutive parameters and object-dependent performance of the cloaking devices. The homogeneous material parameters of the proposed devices significantly facilitate the realization of acoustic external cloaking devices. To give a more realistic point of view, the required materials for cloaking devices are realized with the aid of non-resonant acoustic meta-atoms. Due to these benefits, the proposed structures could find applications in varied scenarios such as making submarines invisible from sonar with non-blinded or fenestrate structures. As a proof-of-concept demonstration, the proposed NCAC design is carried out at a selected frequency. It should be noted that the strategy itself is not dependent on the choice of the frequency and the design can be updated for any desired operating frequency. The impact of loss is minimized due to the non-resonance meta-atom. As the meta-atoms are dispersive, the bandwidth of the device can be limited for a practical design. To increase the bandwidth, achromatic metamaterials with inverse design techniques may be used to decrease the frequency dependence of the effective properties. In addition, based on the effective medium theory, the dimension of meta-atom must be much smaller than the operating wavelength. To apply the concept to higher frequencies, the size of meta-atoms should be decreased which is feasible due to the advanced technology of fabrication. The presented method can also be applied to other acoustic devices, such as acoustic cavities, waveguides, illusion devices, etc., to make them non-closed to outer world that could be useful in future acoustic demands.

## Supplementary Information

Supplementary Information
