# Supplementary material for: Non-closed acoustic cloaking devices enabled by sequential-step linear coordinate transformations
Source: Sci Rep. 2021 Jan 19;11:1845. doi: 10.1038/s41598-021-81331-3 (PMC7815784; doi:10.1038/s41598-021-81331-3)
Supplement: Supplementary file 1 — Supplementary Information [file 41598_2021_81331_MOESM1_ESM.docx]

**Non-Closed Acoustic Cloaking Devices Enabled by Sequential-Step Linear Coordinate Transformations:**

**Supplementary Material**

**Zahra Basiri ^1^, Mohammad Hosein Fakheri^1^, Ali Abdolali ^1,*^ and Chen Shen^2,3^**

^1^ Applied Electromagnetic Laboratory, School of Electrical Engineering, Iran University of Science and Technology,

Tehran, 1684613114, Iran

^2^ Department of Mechanical Engineering, Rowan University, Glassboro, NJ, 08028, USA

^3^ Department of Electrical and Computer Engineering, Duke University, Durham, North Carolina 27708, USA

* [abdolali@iust.ac.ir](mailto:abdolali@iust.ac.ir)

The purpose of this supplementary material is to explain further details of transformation functions that are not presented in the paper.

**Detailed Transformation Function for Region 1**

In figure 2 of the paper, a linear coordinate transformation that maps $\triangle KBE$region in the reference space $\left(x_{1},y_{1}\right)$ to $\triangle KBA$ region in the real space $\left(x_{2},y_{2}\right)$ folds the ground plane boundary $\overline{BE}$ to $\overline{AB}$. In fact, the spoof sound hard boundary or $\triangle KBA$ region, makes an illusionary sound hard boundary condition on $\overline{AB}$ boundary that is shown by red dashed lines also in Fig S1(c).

| 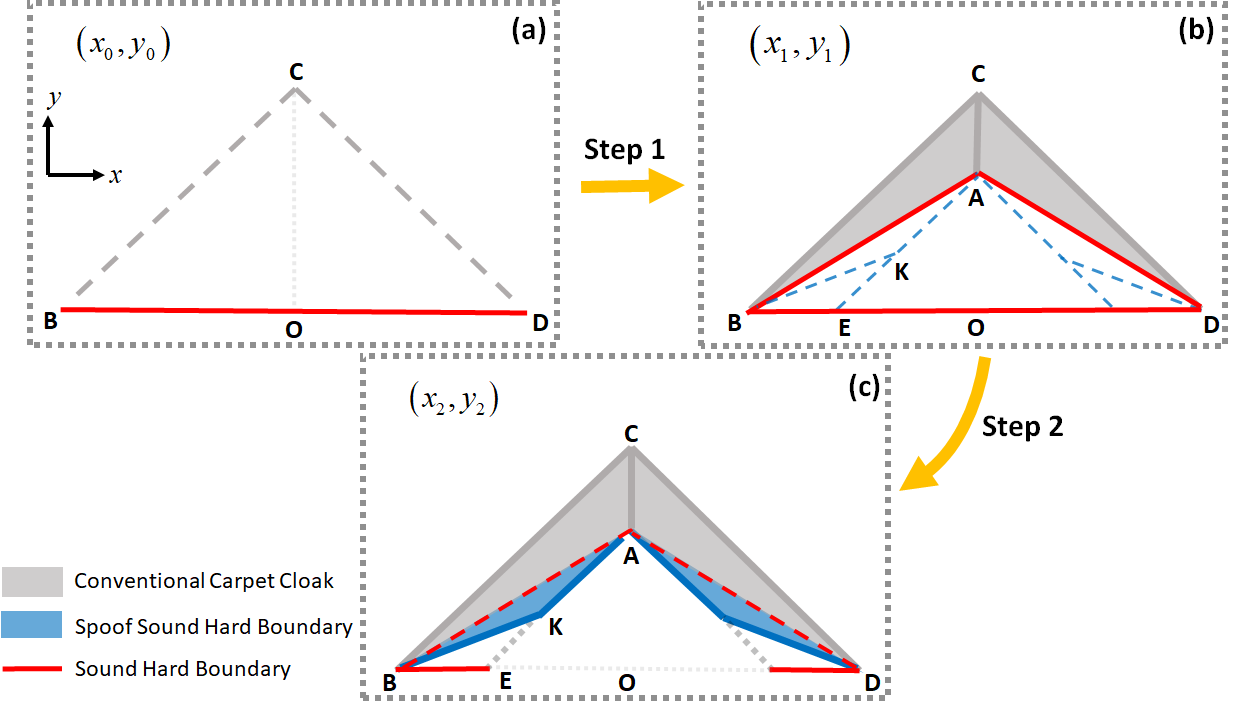 |
| --- |
| **FIG S1. Schematic diagram of first and second steps to NCAC design.** |

For convenience, the left side of the structure is considered and thanks to the symmetry of the structure, all relations can be extended to the right side. For our design, the coordinate values of $O(X_{O},Y_{O})$ , $A(X_{A},Y_{A})$, $C(X_{C},Y_{C})$, $B(X_{B},Y_{B})$, $E(X_{E},Y_{E})$and $K(X_{K},Y_{K})$points in Fig. S1(c) in SI units are $O(0,0)$, $A(0,0.12)$, $C(0,0.25)$, $B(-0.19,0)$, $E(\text{-0.0912},0)$ and $K(\text{-0.0851},0.008)$.

The corresponding transformation equation for the first designing step, which represents the mapping of a reflecting plane in the reference space $\left(x_{0},y_{0}\right)$ to a SHB bump in the real space denoted by $\left(x_{1},y_{1}\right)$ (as depicted in Fig. S1), can be expressed as:

| $\triangle BOC\to\triangle BAC:\begin{matrix} & \begin{matrix} x_{1}=x_{0} \\ y_{1}=\frac{1}{m_{AB}}x_{0}+\left(1-\frac{Y_{A}}{Y_{C}}\right)y_{0}+Y_{A}=0.6316x_{0}+0.52y_{0}+0.12 \end{matrix} \end{matrix}$ | (S1) |
| --- | --- |

wherein $Y_{A}=0.12$and $Y_{C}=0.25$ are the ordinates of $A$ and $C$ points in Fig. S1 and $m_{AB}=0.6316$is the inverted slope of $\overline{AB}$or $\left(X_{A}-X_{B}\right)/\left(Y_{A}-Y_{B}\right)$. Due to the acoustic coordinate transformation theory, constitutive parameters for the resultant conventional cloak are obtained from the Jacobin matrix$J=\partial(x_{1},y_{1})/\partial(x_{0},y_{0})=\left[\begin{matrix} 1 & 0 \\ 0.6316 & 0.52 \end{matrix}\right]$ as follows:

|  | (S2) |
| --- | --- |

wherein $\rho_{0}$ and $\kappa_{0}$ are the mass density and bulk modulus of the host fluid and $J_{0}=Y_{C}/\left(Y_{C}-Y_{A}\right)=1.9231$. In a similar way, for the second step depicted in Fig. S1(a, b), the transformation function related with the SSHB region is given by:

| $\triangle KBE\to\triangle KBA:\begin{matrix} & \begin{matrix} x_{2}= 1.9231x_{1}\text{-12.1015}y_{1}+0.1754 \\ y_{2}=1.2146x_{1}\text{-14.9231}y_{1}+0.2308 \end{matrix} \end{matrix}$ | (S3) |
| --- | --- |

The above transformation function folds the $\triangle KBE$region in the reference space $\left(x_{1},y_{1}\right)$ to the $\triangle KBA$ region in the real space $\left(x_{2},y_{2}\right)$and makes an illusionary SHB on the $\overline{AB}$ boundary as illustrated in Fig. 2(c). By employing the corresponding Jacobin matrix $J=\partial(x_{2},y_{2})/\partial(x_{1},y_{1})=\left[\begin{matrix} 1.9231 & \text{-12.1015} \\ 1.2146 & \text{-14.9231} \end{matrix}\right]$, the constitutive parameters of the SSHB region are obtained as:

|  | (S4) |
| --- | --- |

Then, corresponding to the third step illustrated in Fig. 2S(a, b), the transformation equation that maps the carpet cloak with SSHBs in the reference space $\left(x_{2},y_{2}\right)$ to non-closed compressed regions denoted by region 1 in the real space $\left(x_{3},y_{3}\right)$ is expressed as follows:

| $\triangle OCB\to\triangle OC'B:\begin{matrix} & \begin{matrix} x_{3}=x_{2}+km_{OC'}y_{2}=x_{2}\text{-0.2461}y_{2} \\ y_{3}=ky_{2}=0.6762y_{2}\begin{matrix} \begin{matrix} & & \end{matrix} & \end{matrix} \end{matrix} \end{matrix}$ | (S5) |
| --- | --- |

| 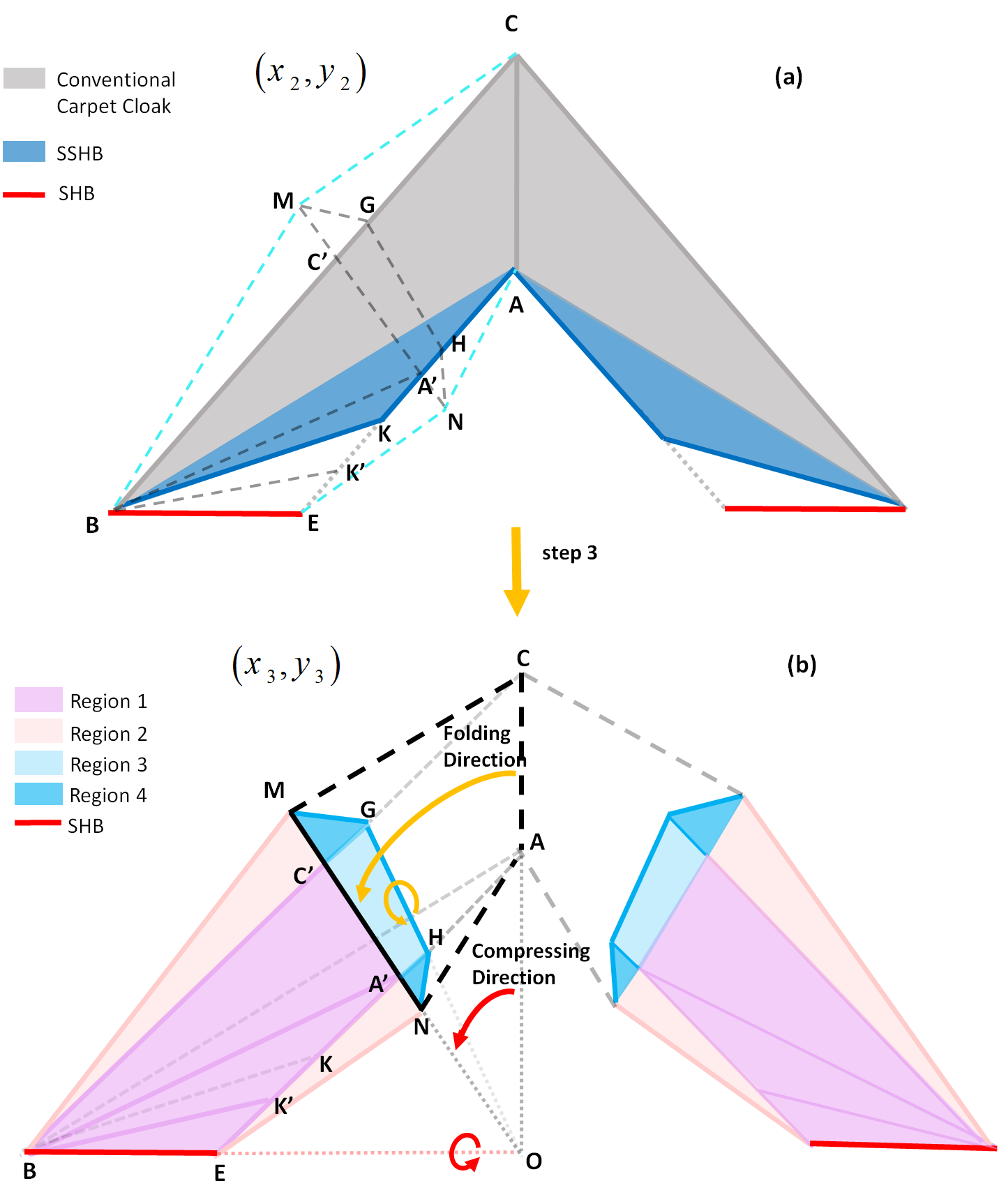 |
| --- |
| **FIG S2. Schematic diagram of the proposed object independent non-closed carpet cloak.** |

In the equation S5, $k=\frac{Y_{C'}}{Y_{C}}=0.6762$, represents the compressing ratio. By applying above coordinate transformation on carpet cloak with SSHBs, the coordinate values for transformed points of $A'(X_{A'},Y_{A'})$, $C'(X_{C'},Y_{C'})$ , $K'(X_{K'},Y_{K'})$,$M(X_{M},Y_{M})$, $N(X_{N},Y_{N})$,$G(X_{G},Y_{G})$and $H(X_{H},Y_{H})$ are respectively calculated as$A'(\text{-0.0295},0.0811)$, $C'(\text{-0.0615},0.169)$, $K'(\text{-0.0871},0.0054)$, $M(\text{-0.0932},0.2560)$ , $N(\text{-0.0151},0.0416)$, $G(\text{-0.0358},0.2029)$and $H(\text{-0.0172},0.0974)$ in SI units. The whole compressed structure is depicted in Fig. S2(b).

The transformation function in equation S5, finally gives the material properties of each different part of region 1 (Fig. S2(b)) as:

|  | (S6) |
| --- | --- |

wherein $m_{OC}=\left(X_{O}-X_{C}\right)/\left(Y_{O}-Y_{C}\right)=0$and $m_{OC'}=\left(X_{O}-X_{C'}\right)/\left(Y_{O}-Y_{C'}\right)=\text{-0.3640}$are the inverted slopes of $\overline{OC}$and $\overline{OC'}$, respectively. In addition,,$\kappa_{cloak}=1.9231\kappa_{0}$, and $\kappa_{SSHB}\text{=-0.0714}\kappa_{0}$, are material properties of carpet cloak and SSHB before compression.

**Detailed Transformation Function for Region 2**

Similarly, the compression transformation functions are applied to the surrounding host fluid in order to achieve region 2 illustrated in Fig. S2(b). The $\triangle BMC$ in the reference space $\left(x_{2},y_{2}\right)$ is compressed to $\triangle BMC'$in the real space $\left(x_{3},y_{3}\right)$ with the transformation function:

| $\triangle BMC\to\triangle BMC':\begin{matrix} & \begin{matrix} \begin{matrix} x_{3}=\underbrace{\left(\frac{X_{B}Y_{M}-X_{B}Y_{C}+X_{M}Y_{C}-X_{C'}Y_{M}}{\Delta_{0}}\right)}_{a_{0}}x_{2}+\underbrace{\left(\frac{X_{M}X_{C'}-X_{B}X_{C'}}{\Delta_{0}}\right)}_{b_{0}}y_{2}\begin{matrix} \begin{matrix} \begin{matrix} \begin{matrix} & \end{matrix} & \begin{matrix} \end{matrix} \end{matrix} & \end{matrix} & \end{matrix} \\ \begin{matrix} & \end{matrix}+\underbrace{\left(\frac{X_{B}X_{C'}Y_{M}-X_{B}X_{C}Y_{M}}{\Delta_{0}}\right)}_{c_{0}}=0.3554x_{2}+0.2438y_{2}\text{-0.1225} \end{matrix} \\ \begin{matrix} y_{3}=\underbrace{\left(\frac{Y_{M}Y_{C}-Y_{M}Y_{C'}}{\Delta_{0}}\right)}_{d_{0}}x_{2}+\underbrace{\left(\frac{X_{B}Y_{M}-X_{B}Y_{C'}+X_{M}Y_{C'}}{\Delta_{0}}\right)}_{e_{0}}y_{2}+\underbrace{\left(\frac{X_{B}Y_{M}Y_{C'}-X_{B}Y_{M}Y_{C}}{\Delta_{0}}\right)}_{f_{0}} \\ =\text{-0.8482}x_{2}+1.3208y_{2}\text{-0.1612} \end{matrix} \end{matrix} \end{matrix}$ | (S7) |
| --- | --- |

wherein $\Delta_{0}=X_{B}Y_{M}-X_{B}Y_{C}+X_{M}Y_{C}-X_{C}Y_{M}=\text{ -0.0244}$. In a similar manner, $\triangle ENA$ in the reference space $\left(x_{2},y_{2}\right)$ is also compressed to $\triangle ENA'$ in the real space $\left(x_{3},y_{3}\right)$ by following coordinate transformation:

| $\triangle ENA\to\triangle ENA':\begin{matrix} & \begin{matrix} \begin{matrix} x_{3}=\underbrace{\left(\frac{-X_{E}Y_{A}+X_{N}Y_{A}-X_{A'}Y_{N}+X_{E}Y_{N}}{\Delta_{1}}\right)}_{a_{1}}x_{2}+\underbrace{\left(\frac{X_{N}X_{A'}-X_{E}X_{A'}}{\Delta_{1}}\right)}_{b_{1}}y_{2}+\underbrace{\left(\frac{X_{E}X_{A'}Y_{N}}{\Delta_{1}}\right)}_{c_{1}} \\ \begin{matrix} & = \end{matrix}1.2304x_{2}\text{-0.4212}y_{2}+0.0210 \end{matrix} \\ \begin{matrix} y_{3}=\underbrace{\left(\frac{Y_{N}Y_{A'}-Y_{N}Y_{A}}{\Delta_{1}}\right)}_{d_{1}}x_{2}+\underbrace{\left(\frac{X_{N}Y_{A'}-X_{E}Y_{A'}+X_{E}Y_{N}}{\Delta_{1}}\right)}_{e_{1}}y_{2}+\underbrace{\left(\frac{X_{E}Y_{A'}Y_{N}-X_{E}Y_{A}Y_{N}}{\Delta_{1}}\right)}_{f_{1}} \\ \begin{matrix} & = \end{matrix}0.3032x_{2}\text{+0.4457}y_{2}+0.0277 \end{matrix} \end{matrix} \end{matrix}$ | (S8) |
| --- | --- |

that $\Delta_{1}=X_{N}Y_{A}-X_{E}Y_{A}+X_{E}Y_{N}-X_{N}Y_{E}=0.0053$. According to Eqs. (S7) and (S8), the constitutive materials of $\triangle ENA'$and $\triangle BMC'$ that construct region 2 in Fig. S2(b) are expressed as:

|  | (S9) |
| --- | --- |

**Detailed Transformation Function for Region 3**

Subsequently, the complementary medium $GHA'C'$ denoted by region 3 in Fig. S2(b) is derived by applying the transformation function:

| $\triangle OGC\to\triangle OGC':\begin{matrix} & \begin{matrix} x_{3}=\left(1-k\frac{m_{OC'}}{m_{OG}}\right)x_{2}+km_{OC'}y_{2} \\ y_{3}=\left(\frac{1}{m_{OG}}\left(1-k\right)\right)x_{2}+km_{OC'}y_{2} \end{matrix} \end{matrix}$ | (S10) |
| --- | --- |

whose Jacobin matrix is $J=\left[\begin{matrix} \text{-0.3957} & \text{-0.2461} \\ \text{-1.8365} & 0.6762 \end{matrix}\right]$and folds the $\overline{GH}$ boundary to itself and $\overline{AC}$ to $\overline{A'C'}$. In addition, $m_{OG}=\left(X_{O}-X_{G}\right)/\left(Y_{O}-Y_{G}\right)=\text{-0.1763}$and $m_{OC'}=\left(X_{O}-X_{C'}\right)/\left(Y_{O}-Y_{C'}\right)=\text{-0.3640}$are the inverted slopes of $\overline{OG}$and $\overline{OC'}$, respectively. Eq. (S10) gives the material properties of the polygon region $GHC'A'$ denoted by region 3 in Fig. S2(b) as follows:

|  | (S11) |
| --- | --- |

**Detailed Transformation Function for Region 4**

Finally, transformation equations are applied to the specify region 4 in Fig. S2(b). The $\triangle GMC'$ domain of region 4 in the real space $\left(x_{3},y_{3}\right)$ is determined by the transformation function:

| $\triangle GMC\to\triangle GMC':\begin{matrix} & \begin{matrix} \begin{matrix} x_{3}=\underbrace{\left(\frac{X_{M}Y_{C}-X_{C'}Y_{M}-X_{M}Y_{G}+X_{G}Y_{M}-X_{G}Y_{C}+X_{C'}Y_{G}}{\Delta_{2}}\right)}_{a_{2}}x_{2}+\underbrace{\left(\frac{X_{M}X_{C'}-X_{C'}X_{G}}{\Delta_{2}}\right)}_{b_{2}}y_{2} \\ \begin{matrix} & \end{matrix}+\underbrace{\left(\frac{X_{C'}X_{G}Y_{M}-X_{M}X_{C'}Y_{G}}{\Delta_{2}}\right)}_{c_{2}}=0.2903x_{2}\text{-0.7675}y_{2}+0.1303 \\ \end{matrix} \\ \begin{matrix} y_{3}=\underbrace{\left(\frac{Y_{M}Y_{C}-Y_{M}Y_{C'}-Y_{C}Y_{G}-Y_{C'}Y_{G}}{\Delta_{2}}\right)}_{d_{2}}x_{2}+\underbrace{\left(\frac{X_{M}Y_{C'}-X_{M}Y_{G}+X_{G}Y_{M}-X_{G}Y_{C'}}{\Delta_{2}}\right)}_{e_{2}}y_{2}\begin{matrix} & \end{matrix} \\ \begin{matrix} & \end{matrix}+\underbrace{\left(\frac{X_{M}Y_{C}Y_{G}-X_{G}Y_{M}Y_{C}-X_{M}Y_{C'}Y_{G}+X_{G}Y_{M}Y_{C'}}{\Delta_{2}}\right)}_{f_{2}}=\text{-0.9339}x_{2}\text{-0.0098}y_{2}+0.1715 \end{matrix} \end{matrix} \end{matrix}$ | (S12) |
| --- | --- |

wherein$\Delta_{3}=X_{H}Y_{A}-X_{N}Y_{A}+X_{N}Y_{H}-X_{H}Y_{N}=\text{-0.0046}$. The coordinate transformation described in Eq. (S12) folds the $\overline{MC}$ boundary in the reference space $\left(x_{2},y_{2}\right)$to $\overline{MC'}$ in the real space $\left(x_{3},y_{3}\right)$and also folds the outer boundary $\overline{MG}$to itself. Moreover, the $\triangle HNA'$ domain of region 4 in the real space $\left(x_{3},y_{3}\right)$ is determined by the transformation equation:

| $\triangle HNA\to\triangle HNA':\begin{matrix} & \begin{matrix} \begin{matrix} x_{3}=\underbrace{\left(\frac{-X_{N}Y_{A}+X_{A'}Y_{N}+X_{H}Y_{A}-X_{A'}Y_{H}+X_{N}Y_{H}-X_{H}Y_{N}}{\Delta_{3}}\right)}_{a_{3}}x_{2}+\underbrace{\left(\frac{X_{H}X_{A'}-X_{A'}X_{N}}{\Delta_{3}}\right)}_{b_{3}}y_{2} \\ \begin{matrix} & \end{matrix}+\underbrace{\left(\frac{X_{A'}X_{N}Y_{A}-X_{A'}X_{H}Y_{N}}{\Delta_{3}}\right)}_{c_{3}}=\text{-0.6410}x_{2}\text{-0.0597}y_{2}\text{-0.0224} \\ \end{matrix} \\ \begin{matrix} y_{3}=\underbrace{\left(\frac{-Y_{A}Y_{N}+Y_{A'}Y_{N}+Y_{A}Y_{H}-Y_{A'}Y_{H}}{\Delta_{3}}\right)}_{d_{3}}x_{2}+\underbrace{\left(\frac{-X_{N}Y_{A'}+X_{H}Y_{A'}+X_{H}Y_{N}-X_{H}Y_{N}}{\Delta_{3}}\right)}_{e_{3}}y_{2}\begin{matrix} & \end{matrix} \\ \begin{matrix} & \end{matrix}+\underbrace{\left(\frac{X_{H}Y_{A}Y_{N}-X_{N}Y_{A}Y_{H}+X_{N}Y_{A'}Y_{A}-X_{H}Y_{N}Y_{A'}}{\Delta_{3}}\right)}_{f_{3}}=\text{-2.1592}x_{2}\text{+0.9214}y_{2}\text{-0.0224} \end{matrix} \end{matrix} \end{matrix}$ | (S13) |
| --- | --- |

with the assumption of $\Delta_{2}=X_{M}Y_{C}-X_{M}Y_{G}+X_{G}Y_{M}-X_{G}Y_{C}=\text{-0.0010}$. The coordinate transformation presented in Eq. (S13) folds the $\overline{NA}$ boundary in reference space $\left(x_{2},y_{2}\right)$to $\overline{NA'}$ in the real space $\left(x_{3},y_{3}\right)$and folds the outer boundary $\overline{HN}$ to itself. Eqs. (S12) and (S13) respectively give the material properties of $\triangle GMC'$ and $\triangle HNA'$ domains of region 4 as follows:

|  | (S14) |
| --- | --- |

The presented three design steps illustrated in Figs. S1 and S2 to achieve a non-closed carpet cloak are demonstrated by mathematical terminology (Eq. (S1) to Eq. (S14)). The transformation method can be extended to achieve more number of windows in the structure and it could also be applied to other acoustic devices to make them fenestrated.

To summarize, all above constitutive parameters are presented in Table S1.

**Table S1.** The constitutive materials of the NCAC's regions

| Region 1 |  |
| --- | --- |
|  |
|  |
| Region 2 |  |
|  |
| Region 3 |  |
| Region 4 |  |
|  |
